# Depletion of CD11c^+^ cells in the CD11c.DTR model drives expansion of unique CD64^+^ Ly6C^+^ monocytes that are poised to release TNF-α

**DOI:** 10.1002/eji.201545789

**Published:** 2015-11-30

**Authors:** Shivajanani Sivakumaran, Stephen Henderson, Sophie Ward, Pedro Santos E Sousa, Teresa Manzo, Lei Zhang, Thomas Conlan, Terry K Means, Maud D'Aveni, Olivier Hermine, Marie-Thérèse Rubio, Ronjon Chakraverty, Clare L Bennett

**Affiliations:** 1 Institute for Immunity and Transplantation, University College LondonLondon, UK; 2 Cancer Institute, University College LondonLondon, UK; 3 Bill Lyons Informatics Centre, University College LondonLondon, UK; 4 MGH Center for Immunology and Inflammatory Diseases, Harvard Medical SchoolBoston, MA, USA; 5 CNRS UMR 8147, Université Paris Descartes, Faculté de MédecineHôpital Necker, Paris, France

**Keywords:** CD11c.DTR, Dendritic cells, Depletion, Diphtheria toxin receptor (DTR), Monocytes, Monocytosis

## Abstract

Dendritic cells (DCs) play a vital role in innate and adaptive immunities. Inducible depletion of CD11c^+^ DCs engineered to express a high-affinity diphtheria toxin receptor has been a powerful tool to dissect DC function in vivo. However, despite reports showing that loss of DCs induces transient monocytosis, the monocyte population that emerges and the potential impact of monocytes on studies of DC function have not been investigated. We found that depletion of CD11c^+^ cells from CD11c.DTR mice induced the expansion of a variant CD64^+^ Ly6C^+^ monocyte population in the spleen and blood that was distinct from conventional monocytes. Expansion of CD64^+^ Ly6C^+^ monocytes was independent of mobilization from the BM via CCR2 but required the cytokine, G-CSF. Indeed, this population was also expanded upon exposure to exogenous G-CSF in the absence of DC depletion. CD64^+^ Ly6C^+^ monocytes were characterized by upregulation of innate signaling apparatus despite the absence of inflammation, and an increased capacity to produce TNF-α following LPS stimulation. Thus, depletion of CD11c^+^ cells induces expansion of a unique CD64^+^ Ly6C^+^ monocyte population poised to synthesize TNF-α. This finding will require consideration in experiments using depletion strategies to test the role of CD11c^+^ DCs in immunity.

## Introduction

Dendritic cells (DCs) play a vital role in the activation and control of cellular immunity. Much of our understanding of the requirement for DCs in regulating immunity has come from in vivo models of DC depletion in which different DC populations are engineered to express a high-affinity diphtheria toxin (DT) receptor (DTR), and can be inducibly and transiently depleted in vivo upon injection of DT 1,2. Inducible cell ablation is a powerful tool to understand the role of a population of cells in vivo. However, it is important that conclusions made regarding the physiological functions of DCs following experimental depletion are also informed by the impact that depletion has upon other cells in the immune system.

Bone marrow (BM) derived circulating monocytes can be divided into two populations, which are distinguished in mice by expression of the glycoprotein Ly6C 3,4. “Nonclassical” Ly6C^neg^ monocytes patrol endothelial vessels in the steady state and are positioned to initiate a rapid response to infection 5. “Classical” or “inflammatory” Ly6C^+^ monocytes are released from the BM via ligation of the chemokine receptor CCR2 6–8. The recent identification of a common monocyte progenitor (cMoP) 9 and lineage-tracing studies suggest a linear model in which cMoPs give rise to Ly6C^+^ cells, which in turn seed the Ly6C^neg^ pool 4,10. Activation of Ly6C^+^ monocytes results in the upregulation of molecules associated with macrophage and DC function, and classical monocytes were long thought to function as a pool of rapid reactionary cells capable of augmenting tissue DCs and macrophages during inflammation 11–14. However, a number of recent studies have now demonstrated that Ly6C^+^ monocytes directly participate in innate and adaptive immunities 15–18, and in the absence of infection, monocytes extravasate into tissues and may contribute to immunosurveillance of peripheral antigens 19,20. Migration across the vascular endothelium into tissue is sufficient to induce upregulation of molecules such as the high-affinity IgG receptor FcγRI (CD64), and is linked to transition toward a more activated and/or differentiated cell type 20,21.

The relative proportion of monocytes within the steady-state mononuclear phagocyte system (MPS) is finely controlled by serum levels of myeloid growth factors such as M-CSF, G-CSF, and fms-like tyrosine kinase receptor-3 (ligand, Flt3(L)) 22. This balance has been exemplified in studies using the constitutive and transient depletion of DCs. CD11c.DTA mice that are born lacking DCs develop a myeloproliferative syndrome by 3 months of age due to enhanced extramedullary hematopoiesis in the spleen resulting in accumulation of CD115^+^ monocytes 23. Homeostasis of DC populations depends on signaling via Flt3, and removal of DCs results in increased serum levels of Flt3(L) 23–25. The increase in Flt3(L) drives myeloproliferation in DC-deficient animals, due to the direct stimulation of myeloid progenitors in the spleen 23. Similarly, transient loss of DCs from DC-specific DTR mice also induces monocytosis 2,23,26–28. However, despite reports of enhanced numbers of monocytes in DC-depleted mice the immune characteristics of these cells have not been investigated.

In this study, we find that transient depletion of CD11c^+^ cells from CD11c.DTR mice results in the CCR2-independent, G-CSF-dependent expansion of a novel CD64^+^ Ly6C^+^ monocyte population in the spleen that is transcriptionally distinct from both Ly6C^+^ and Ly6C^neg^ monocytes. This variant monocyte population is poised to release TNF-α on stimulation with LPS, and has the potential to mediate inflammatory immunity in the absence of DCs.

## Results

### Depletion of CD11c^+^ cells leads to expansion of a distinct population of CD64^+^ Ly6C^+^ monocytes

**Figure 1:**
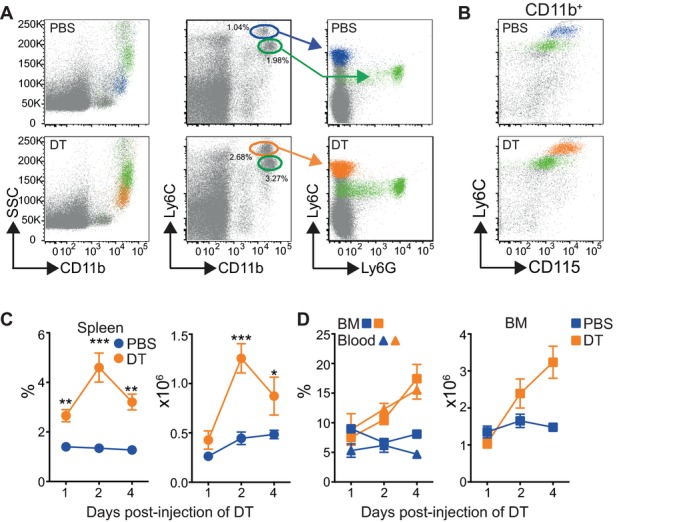
Expansion of Ly6C^+^ monocytes following depletion of CD11c^+^ cells. CD11c.DTR mice were injected with PBS or DT and analyzed 48 h later. (A) Representative dot plots showing the expansion of monocytes (blue and orange) and neutrophils (green) in the spleens of PBS- and DT-injected CD11c.DTR mice. Dot plots are pregated on live single cells. Dot plots on the far right show expression of Ly6C and Ly6G by CD11b^+^Ly6C^high^ (blue and orange) and CD11b^+^Ly6C^int^ (green) gated cells overlaid on live cells. (B) Representative dot plots showing expression of CD115 and Ly6C by gated CD11b^+^Ly6C^high^ (blue and orange) or Ly6C^int^ (green) cells. Plots are representative of more than three independent experiments. (C) Graphs show the percentage ± SEM (left) and number ± SEM (right) of CD11b^+^Ly6C^high^ monocytes in the spleen of PBS-treated (blue) and DTR-treated (orange) recipients at different times post injection of DT. Two-way ANOVA with multiple comparisons (*) PBS versus DT on day 2 *p* < 0.0001. Data are pooled from multiple independent experiments: day 1 PBS/DT *n* = 6, day 2 PBS/DT *n* = 26/24, day 4 PBS/DT *n* = 6. (D) Left: graph showing the frequency ± SEM of monocytes in the blood and BM of PBS-treated (blue triangles and squares) and DT-treated (orange triangles and squares) recipients. Two-way ANOVA with multiple comparisons (*) day 2 BM and blood *p* = 0.0016 and *p* = 0.0012, respectively, day 4 *p* < 0.0001 for both. Right: graph showing the number ± SEM of monocytes in the BM from the femurs and tibias of one back leg. Two-way ANOVA with multiple comparisons (*) *p* = 0.0015 on day 4. Data are pooled from multiple independent experiments: days 1 and 4 PBS/DT *n* = 6, day 2 BM PBS/DT *n* = 14/12, blood PBS/DT *n* = 12.

To investigate the effect of depletion of CD11c^+^ DCs on the MPS, we injected CD11c.DTR/EGFP mice with a single dose of DT or PBS, and analyzed myeloid cells in the spleen 48 h later. Supporting Information Figure 1 shows that CD11c^high^ GFP^+^ DCs are efficiently ablated from the spleen in this model, along with CD11b^+^F4/80^+^Ly6C^low^ macrophages as has been previously reported 28,29. Other nonmyeloid immune populations were not affected (Supporting Information Fig. 1C). Analysis of splenocytes from DT-treated recipients demonstrated that depletion of CD11c^+^ cells resulted in the expansion of two populations of granular side scatter (SSC)^int to high^ CD11b^+^ myeloid cells, in accord with published data 26,30,31, that differentially expressed Ly6C (Fig. 1A). Further, flow cytometric analyzes defined SSC^int^ cells as Ly6C^high^CD115^high^ classical monocyte-like cells (referred to as Ly6C^+^ monocytes). The second SSC^high^ population was designated CD11b^+^Ly6C^+^CD115^int^ neutrophils, which were further defined by expression of Ly6G (Fig. 1A and B). Staining with Ly6G also highlighted the presence of an uncharacterized population of Ly6G^neg^ Ly6C^int^ cells in DT-treated mice (Fig. 1A). Kinetic analyzes demonstrated that the increase in splenic Ly6C^+^ monocytes peaked 2 days after the injection of DT and decreased by day 4 (Fig. 1C). We also observed a concurrent peak in the number of splenic neutrophils (Supporting Information Fig. 2A). However, in the blood and BM, monocytes continued to expand over the course of the experiment (Fig. 1D). By contrast, the frequency of neutrophils rapidly increased in the blood and BM 1 day after treatment. This increase was not matched by a change in the numbers of BM neutrophils, possibly due to small differences in the frequency of BM neutrophils in the context of other changing cell populations (Supporting Information Fig. 2B). The increases in splenic monocytes and neutrophils were also confirmed by analyzing expansion of CD11b^+^Ly6C^+^ and Ly6G^+^ cells, respectively, 48 h after the injection of PBS or DT (Supporting Information Fig. 2C). To confirm that expansion of Ly6C^+^ cells was dependent on depletion of CD11c-expressing cells, we injected DT into both wild-type (WT) C57BL/6 mice and Langerin.DTR mice, the latter another DTR expressing strain, and analyzed the expansion of Ly6C^+^ cells in the spleen 48 h later. Ly6C^+^ cells did not expand in the spleen or epidermis of either DT-treated WT or Langerin.DTR mice (Supporting Information Fig. 2D). Together, these data suggest that the loss of CD11c^+^ cells drives expansion of monocytes and neutrophils in the spleen of CD11c.DTR mice. These observations agree with other published studies that have reported monocytosis and neutrophilia after depletion of DCs in other, more specific, models of DC depletion 27,28,31,32. In particular, depletion of DCs, but not macrophages, results in monocytosis in zDC.DTR mice 27. Therefore, together these studies strongly suggest that the loss of DCs is sufficient to drive expansion of myeloid cells in these mice.

**Figure 2:**
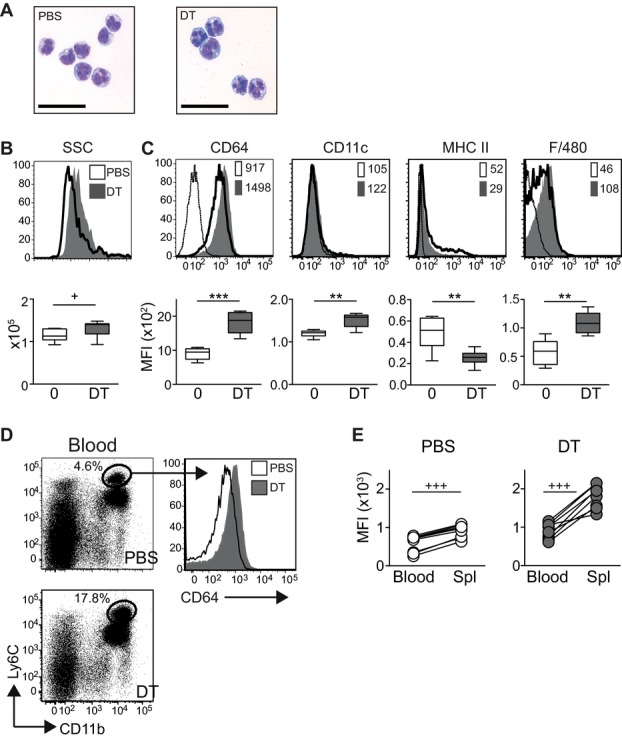
DT-Ly6C^+^ monocytes express CD64. (A) Photomicrographs show Giemsa-stained cytospins of sorted CD11b^+^Ly6C^high^CD115^high^ cells from PBS- and DT-treated mice, bars = 100 μM. (B) Representative histograms (top) and graph (bottom) showing SSC of CD11b^+^Ly6C^high^CD115^high^ monocytes from PBS-treated (white) or DT-treated (gray) mice. Graph shows SSC ± SEM from PBS-treated (*n* = 15) and DT-treated (*n* = 13) mice. Data are pooled from four independent experiments: PBS versus DT *p* = 0.0186. (C) Top: representative histograms showing expression of labeled surface markers on CD11b^+^Ly6C^high^ or CD11b^+^Ly6C^high^CD115^high^ monocytes from PBS-treated (white) and DT-treated (gray) mice. Staining with the relevant isotype control is shown by the thin line. Numbers are the median fluorescence intensity (MFI). Bottom: Graphs show the MFI ± SEM for CD64, CD11c, MHC II, and F4/80 on monocytes gated as for the histograms (*n* = 8 or 6 for F4/80 expression). Horizontal lines mark the mean; data are pooled from three independent experiments: CD64 PBS versus DT *p* = 0.0002, CD11c *p* = 0.0014, MHC II *p* = 0.0070, F4/80 *p* = 0.0043. (D) Left: representative dot plots showing the frequency of gated live cells in the blood of CD11c.DTR mice injected with DT 48 h earlier. Right: histograms showing expression of CD64 by CD11b^+^Ly6C^high^ monocytes from PBS-treated (white) and DT-treated (gray) mice. (E) Paired line graphs showing the median fluorescent intensity of CD64 on CD11b^+^Ly6C^high^ monocytes from the blood and spleens of PBS- and DT-treated mice. Data are pooled from three independent experiments, *n* = 8. Student's paired *t*-test in both PBS- and DT-treated animals, *p* < 0.0001. Statistical analyses for other experiments were carried out with a Student's unpaired *t*-test (+) or Mann–Whitney (*) as appropriate.

To compare monocytes from PBS- or DT-injected mice, we initially examined the cells by microscopy. Ly6C^+^ monocytes in DT-injected mice were more vacuolated than monocytes from PBS-injected controls, suggesting that the depletion of DCs results in the expansion of a more differentiated cell (Fig. 2A). Therefore, we analyzed features associated with monocyte differentiation in vivo. Figure 2B shows that Ly6C^+^ monocytes from DT-injected mice were more granular (increased SSC) than cells from control mice. Both populations expressed low-to-negative levels of CD11c, MHC II, and F4/80 as described for Ly6C^+^ monocytes 33, although F4/80 was slightly increased on monocytes from DT-treated mice compared with isotype controls. However, the IgG receptor FcγRI (CD64) was expressed in both groups and the level of expression was doubled on DT-Ly6C^+^ cells (Fig. 2C, mean expression on PBS cells = 897.4 ± 59.38 (SEM) compared with 1805 ±108.8 on DT-Ly6C^+^ cells). Circulating Ly6C^+^ cells in the blood of DT-injected mice also expressed higher levels of CD64 than PBS-treated controls (Fig. 2D), but these levels were lower than on Ly6C^+^ monocytes in the spleen (Fig. 2E). Injection of DT did not lead to an increase in tissue monocytes, which expressed the highest levels of CD64 as previously reported (Supporting Information Fig. 3) 20. Collectively, our data demonstrate that the depletion of DCs results in the accumulation of a distinct population of CD64^+^ Ly6C^+^ cells.

### DC depletion drives expansion of a tran-scriptionally distinct population of Ly6C^+^ monocytes

**Figure 3:**
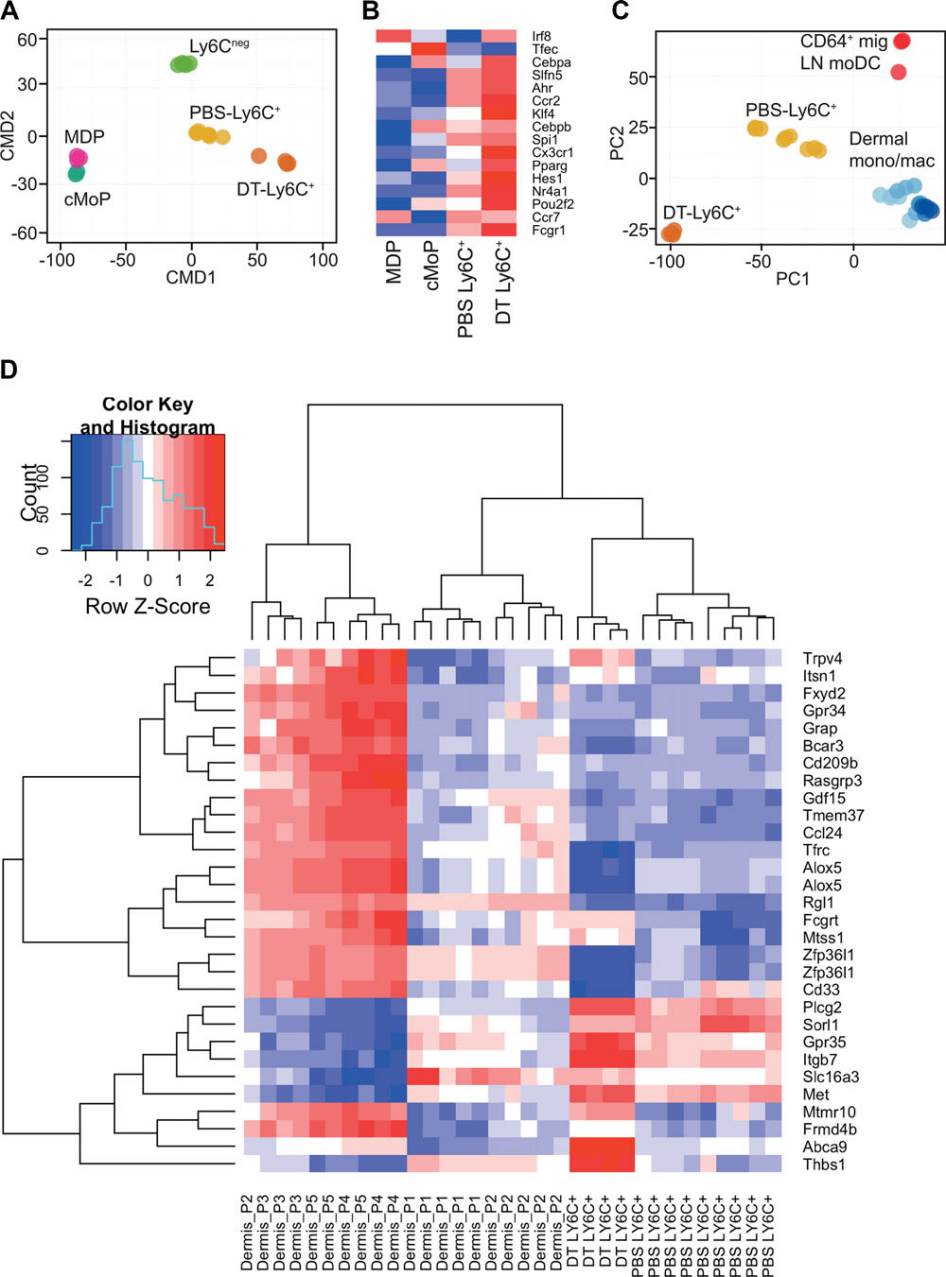
DT-Ly6C^+^ monocytes are transcriptionally distinct from blood and tissue monocyte populations. CD11c.DTR mice were injected with PBS or DT, and 48 h later splenic lineage^neg^CD11b^+^CD115^high^Ly6C^high^ monocytes were sorted to high purity and analyzed by hybridization to an Affymetrix microarray. We also sorted lineage^neg^CD11b^+^CD115^high^Ly6C^neg^ monocytes and BM cMoPs (lineage^neg^CD117^+^CD115^+^CD135^neg^Ly6C^+^CD11b^neg^) from C57BL/6 mice. (A) Multidimensional Scaling (MDS) comparing the transcriptional profile of DT-Ly6C^+^ cells to other precursors and monocyte populations (Immgen MDP *n* = 3, UCL cMoP *n* = 3, UCL PBS Ly6C^+^ *n* = 4, and Immgen (PBS) Ly6C^+^ *n* = 3, UCL Ly6C^neg^ *n* = 3, and Immgen *n* = 2). Each subset of cells was purified from at least two independent experiments and was derived from individual mice, or pooled from multiple mice. (B) Heat map comparing the relative expression of a panel of genes associated with monocyte differentiation in the different cell populations. (C) PCA of gene expression by DT- and PBS-Ly6C^+^ monocytes in comparison to published data for CD64^+^ migrating LN monocyte-derived DCs and dermal-cell subsets 20. Approximately, 45 and 13% of the variance within the expression data is orthogonal to PC1 and PC2, respectively (58% cumulative). (D) Heat map depicting the relative expression across all samples of the principal gene signature (PC1, Supporting Information Fig. 2D) that separates the dermal P1–P5 group. DT-Ly6C^+^ *n* = 4, PBS-Ly6C^+^ *n* = 9 (Immgen, UCL), P1–P5 20.

Ly6C^+^ MHC class II^+^ blood monocytes were recently identified that seed tissue monocyte populations in the steady state 19]. These cells upregulate CD64 and follow extravasation. Therefore, we examined the relationship between DT-Ly6C^+^ monocytes and other splenic and tissue monocyte populations in more detail. We sorted test and control cell populations to high purity and compared their transcriptional profiles by hybridization to an Affymetrix microarray. Specifically, we purified spleen lineage^neg^CD11b^+^CD115^high^Ly6C^high^ monocytes from DT- or PBS-injected CD11c.DTR mice, spleen lineage^neg^CD11b^+^CD115^high^Ly6C^neg^ monocytes, and BM cMoPs (lineage^neg^CD117^+^CD115^+^CD135^neg^Ly6C^+^CD11b^neg^) 9. All control populations were validated against publically available Immgen datasets (http://www.immgen.org, see materials and methods). Multidimensional scaling analysis, which offers a visual representation of the proximity of the datasets, demonstrated that DT-Ly6C^+^ monocytes were transcriptionally distinct from classical Ly6C^+^ and Ly6C^neg^ monocytes, and were more distant from precursor cells than both these populations, suggesting the development of a more differentiated cell type (Fig. 3A). To explore the lineage relationship between PBS- and DT-Ly6C^+^ monocytes further, we specifically analyzed the expression of a panel of genes that encode transcriptional factors implicated in monocyte differentiation 3,9. The heat map in Figure 3B shows the relative expression of the panel of genes by macrophage/DC precursors (MDPs), cMoPs, and Ly6C^+^ monocytes from PBS- and DT-treated mice. These data demonstrate that DT-Ly6C^+^ monocytes express transcription factors associated with monocyte differentiation at higher levels than the other cell populations, reinforcing the concept that the depletion of DCs drives the expansion of a highly differentiated monocyte population.

DT-Ly6C^+^ cells were transcriptionally distinct from classical splenic Ly6C^+^ monocytes and showed features linked to more advanced differentiation, therefore, we next queried whether they were instead more similar to tissue monocytes. Thus, we compared PBS- and DT-Ly6C^+^ cells with the CD24^neg^ dermal monocyte/macrophage populations and migrating LN CD64^+^ monocyte-derived DCs that can be distinguished on the basis of surface expression of CD64 and MHC Class II (P1–P5) as recently published by Tamoutounour et al. 20. The principal components analysis (PCA) shown in Figure 3C demonstrates that extravasation of Ly6C^+^ monocytes, and differentiation into tissue macrophages, results in the expression of a shared transcriptional profile that was not expressed by either PBS-Ly6C^+^ or DT-Ly6C^+^ monocytes from the spleen. However, DT-Ly6C^+^ monocytes were clearly also distinct from PBS-Ly6C^+^ monocytes according to both principal components 1 and 2. To focus more closely on the differences between the cell types, we examined the signatures that differentiate P1-P5 cells, again using PCA (Supporting Information Fig. 4). Then, using the strongest signature of difference within P1–P5 (the primary principal component PC1) we defined a gene signature that best distinguishes P1/2 from P3–P5. Using this signature, we plotted a heat map displaying this signature across P1-P5 and DT-Ly6C^+^ monocytes (Fig. 3D). The heat map shows that, despite increased expression of CD64, DT-Ly6C^+^ monocytes cluster as a true variant of Ly6C^+^ monocytes and both populations are transcriptionally distinct from cells that have exited the vasculature and entered the tissues. However, consistent with our phenotypic analyzes, DT-Ly6C^+^ monocytes are most similar to the P1/P2 cells of the dermal populations, that is, cells that have recently entered the skin. Of the signature genes that differentiate P1/2 dermal monocytes, DT-Ly6C^+^ cells have upregulated higher levels of expression of a number of genes associated with cell migration and angiogenesis (*Gpr35*, *Itgb7*, *Met*, *Thbs1* 34–37), but significantly downregulated *Cd33*, which has been shown to repress monocyte-derived pro-inflammatory cytokines 38. Thus, our phenotypic and transcriptional profiling data show that the depletion of DCs promotes the expansion of differentiated monocytes that have upregulated expression of CD64, but that remain transcriptionally distinct from tissue monocytes, and are a true variant of splenic classical Ly6C^+^ cells.

### Expansion of Ly6C^+^ monocytes in DC-depleted mice does not require signaling via CCR2

**Figure 4:**
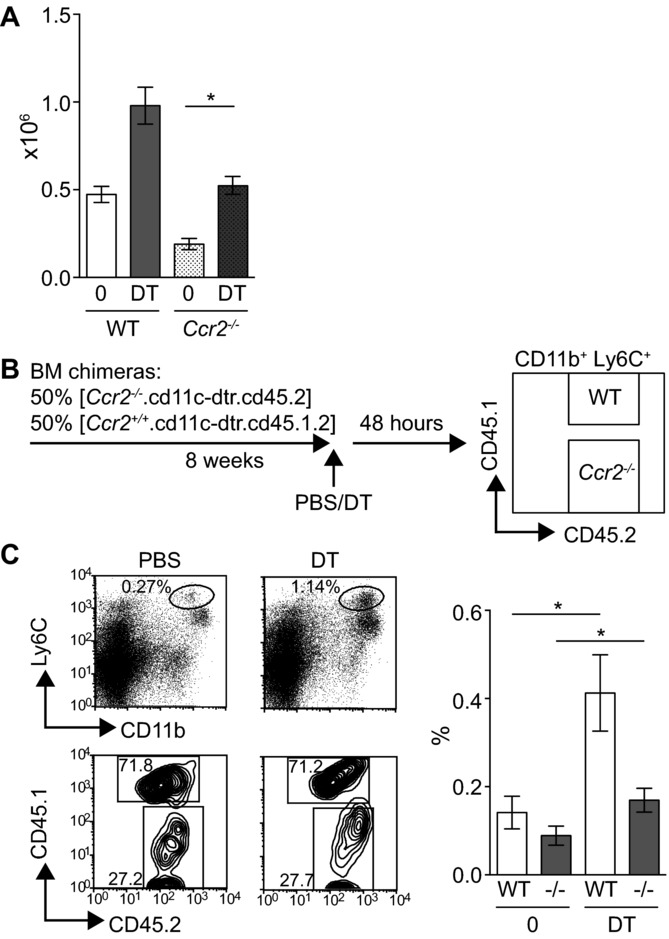
CCR2-independent expansion of DT-Ly6C^+^ monocytes in vivo. (A) CD11c.DTR.Ccr2^−/−^ mice or CD11c.DTR controls were injected with PBS (*n* = 3–4) or DT (*n* = 2–4) and the expansion of monocytes analyzed in the spleen 48 h later. Graph shows the number of CD11b^+^Ly6C^+^ cells ± SEM, PBS versus DT in CD11c.DTR.Ccr2^−/−^ mice *p* = 0.029. Data are pooled from three independent experiments. (B) Schematic diagram describing the competitive chimera experiments. (C) Left: representative flow cytometry plots showing CD11b^+^Ly6C^+^ monocytes in pregated bulk splenic CD45.2^+^ hematopoietic cells from PBS- or DT-treated chimeras, and the percentage of WT (CD45.1^+^CD45.2^+^) or Ccr2^−/−^ (CD45.2^+^) cells in the monocyte gate. Right: graph shows the frequency of WT or Ccr2^−/−^ CD11b^+^Ly6C^+^ cells in the spleens of PBS- or DT-treated chimeras; PBS-treated (*n* = 6) and DT-treated (*n* = 6) mice. WT cells PBS versus DT *p* = 0.0260, Ccr2^−/−^ cells *p* = 0.0411. Data are pooled from two independent experiments. Statistical analyses were carried out with a Mann–Whitney *U* test.

Classical Ly6C^+^ monocytes differentiate from committed precursors in the BM before egress into the blood. Injection of DT increased the frequency of Ly6C^+^ monocytes in the BM, whereas the frequency of macrophage/DC precursors (MDPs) or cMoPs was unchanged (Fig. 1 and Supporting Information Fig. 5A). To determine whether splenic Ly6C^+^ monocytes were directly recruited from the BM, we initially analyzed the expansion of monocytes in mice lacking CCR2. In these mice, monocytes cannot efficiently leave the BM and *Ccr2*^−/−^ animals have decreased numbers of Ly6C^+^ monocytes in the circulation 7,39. Figure 4A shows that the injection of DT into *Ccr2^−^*^/−^.CD11c.DTR mice resulted in an expansion of splenic Ly6C^+^ monocytes to a similar extent as *Ccr2^+/+^*.CD11c.DTR controls (Fig. 4A: *Ccr2^+/+^*.CD11c.DTR PBS- compared to DT-injected mice = 2.1-fold mean increase in the frequency of CD11b^+^Ly6C^+^ monocytes; *Ccr2^−^*^/−^.CD11c.DTR PBS- versus DT-treated = 2.7-fold increase), despite a lack of circulating monocytes in the blood (Supporting Information Fig. 5B). To directly compare the contribution of cells that could or could not signal via CCR2 to monocytosis in DT-treated mice, we adopted a mixed chimera approach 39,40. CD11c.DTR.CD45.1 recipients were lethally irradiated and reconstituted with an equal mixture of BM cells isolated from *Ccr2*^−/−^.CD11c.DTR.CD45.2 or *Ccr2*^+/+^.CD11cDTR.CD45.1.2 donors. Eight weeks later, chimeras were injected with DT, and expansion of *Ccr2*^−/−^ (CD45.2) versus WT (CD45.1.2) CD11b^+^Ly6C^+^ monocytes compared in the blood and spleen (Fig. 4B). As expected, Ly6C^+^ monocytes derived from WT BM preferentially accumulated in the spleen of both PBS- and DT-treated chimeras, but with a significant contribution from *Ccr2^−/−^* cells reflecting the mix of blood-derived and extramedullary monocytes at this site 41. Figure 4C shows that DT treatment of mixed chimeras induced expansion of both WT and *Ccr2^−/−^* Ly6C^+^ monocytes in the spleen (2.9-fold increase in WT cells on injection of DT compared with 1.9-fold increase in *Ccr2*^−/−^ cells). Together, these data show that the depletion of DCs results in the CCR2-independent expansion of some Ly6C^+^ monocytes in the spleen, suggesting that these cells are not recruited from the BM.

### Depletion of DC results in the G-CSF-dependent expansion of Ly6C^+^ monocytes

**Figure 5:**
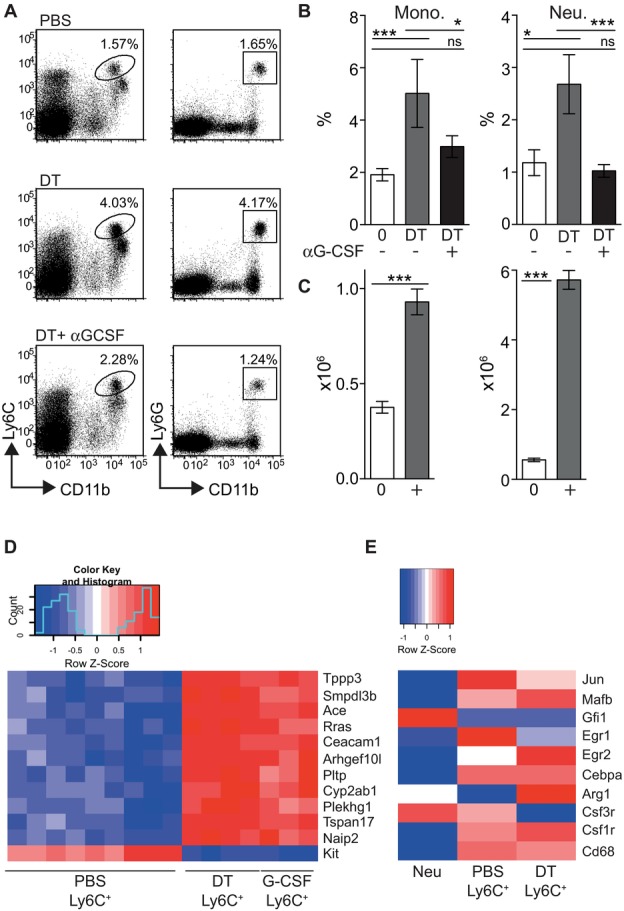
DC-dependent monocytosis requires G-CSF. (A) CD11c.DTR mice were injected with αG-CSF or control prior to the depletion of DCs and analyzed 48 h later. Representative dot plots showing the frequency of gated monocytes or neutrophils in the spleen. Dot plots are pregated on live single cells. (B) Graphs showing the percentage ± SEM of CD11b^+^Ly6C^high^ monocytes (top) or CD11b^+^Ly6G^+^ neutrophils (bottom) in the spleen and blood of PBS-treated (*n* = 8), DT + control-treated (*n* = 9), and DT +  αG-CSF-treated groups (*n* = 12). Splenic monocytes PBS versus DT *p* < 0.001, DT versus DT +  αG-CSF *p* = 0.033, PBS versus DT +  αG-CSF *p* = 0.132; splenic neutrophils PBS versus DT *p* = 0.028, DT versus DT +  αG-CSF *p* < 0.001, PBS versus DT +  αG-CSF *p* = 0.296; ns = not significant. Data are pooled from three independent experiments. (C) WT mice were injected s.c. with PBS (0) or G-CSF (+) for 5 days. Graphs show the number ± SEM of CD11b^+^Ly6C^+^ monocytes (left) and CD11b^+^Ly6G^+^ neutrophils (right) in the spleens of treated or control mice. Cells were gated from plots equivalent to those shown in Figure 1A. Monocytes and neutrophils PBS versus G-CSF *p* < 0.0001. Statistical analyses were carried out with a Mann–Whitney *U* test. (D) Splenic lineage^neg^CD11b^+^Ly6C^+^ monocytes were purified from mice treated with G-CSF (G-CSF-Ly6C^+^ cells). A representative panel of the 12 most significant expression differences between PBS Ly6C^+^ and DT Ly6C^+^ monocytes was elected using the limma procedure. The heat map shows that the G-CSF Ly6C^+^ samples show a strongly similar expression pattern to DT Ly6C^+^ according to these genes: PBS-Ly6C^+^ *n* = 9, DT-Ly6C^+^ *n* = 4, G-CSF-Ly6C^+^ *n* = 3. (E) Heat map showing differential *Z*-scored expression of a neutrophil/monocyte gene panel in the three sets of samples: Neu (*n* = 4), PBS Ly6C^+^ (*n* = 9), and DT Ly6C^+^ (*n* = 4). The gene expression profile for neutrophils was obtained from the Immgen database.

It has been previously reported that the depletion of DCs results in increased serum levels of G-CSF 26,31. To test whether G-CSF was required for the expansion of monocytes in DC-depleted mice, we injected CD11c.DTR mice with αG-CSF neutralizing antibodies prior to injection of PBS or DT. Figure 5A and B demonstrates that endogenous G-CSF was required for the expansion of DT-Ly6C^+^ monocytes and Ly6G^+^ neutrophils in the spleen of DC-depleted mice. Consistent with the hypothesis that an increase in serum growth factors promotes the expansion of splenic monocytes in DC-depleted animals 23, subcutaneous treatment of non-DC-depleted WT mice with exogenous G-CSF also mobilized splenic monocytes and neutrophils but did not lead to an increase in the numbers of these cells in the BM (Fig. 5C; number of CD11b^+^Ly6C^high^Ly6G^neg^ monocytes in the BM of PBS-treated mice = 1.28 × 10^6^ ± 0.1376 × 10^6^ (SEM) compared with 0.89 × 10^6^ ± 0.09712 × 10^6^ in G-CSF-treated mice, *n* = 8 mice per group in two independent experiments). Ly6C^high^ monocytes isolated from the spleens of G-CSF-treated mice were transcriptionally indistinguishable from DT-Ly6C^+^ monocytes, when comparing a panel of genes with the topmost significant expression differences between PBS- and DT-Ly6C^+^ cells (Fig. 5D).

It has been recently shown that murine neutrophils can transdifferentiate into monocytes in vivo 42,43. Therefore, we questioned whether DT-Ly6C^+^ monocytes were differentiating from the enlarged neutrophil population in DC-depleted mice. To address this question, we analyzed the expression profile of a panel of signature neutrophil- and monocyte-associated genes in DT-Ly6C^+^ monocytes 43. The heat map in Figure 5E shows that DT-Ly6C^+^ cells expressed a gene signature consistent with monocytic cells (e.g., *mafB*, *csf1r*), and did not express neutrophil-associated genes (e.g., *gfi1*). Despite the requirement for G-CSF shown in Figure 5A, these analyses revealed that DT-Ly6C^+^ monocytes expressed relatively lower levels of the G-CSF receptor (*csfr3*) than PBS-Ly6C^+^ cells. However, it has been previously shown that the G-CSF receptor is not required for G-CSF-dependent recruitment of neutrophils from the BM, suggesting indirect actions of G-CSF on target cells 44. Together with published data, these results suggest that increases in serum G-CSF are sufficient to drive the expansion of Ly6C^+^ monocytes in DC-depleted mice 26.

### Depletion of DC results in the accumulation Ly6C^+^ monocytes poised for innate immune activation

**Figure 6:**
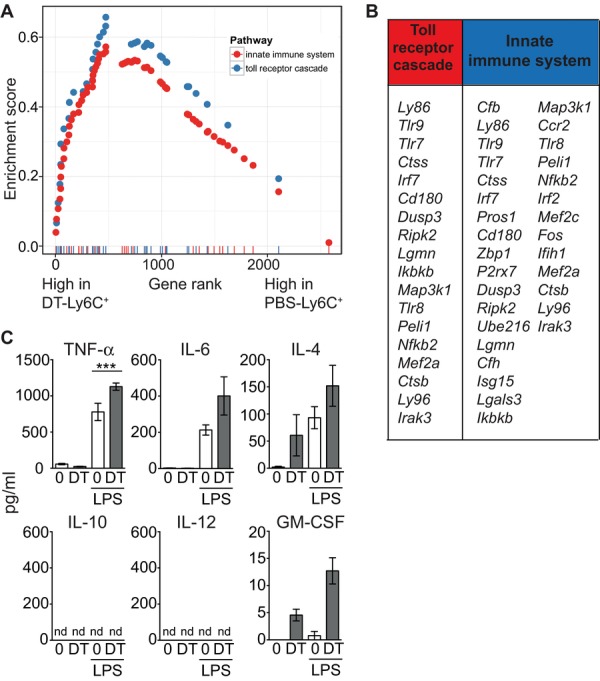
DT-Ly6C^+^ monocytes express genes associated with innate immune activation, and secrete high levels of TNF-α upon stimulation with LPS. (A) Gene set enrichment analysis was used to identify differentially expressed pathways between DT-Ly6C^+^ cells and their most closely related group: PBS-Ly6C^+^. The rank-versus-ES score plots are shown for two representative pathways upregulated in DT-Ly6C^+^ cells: blue = Reactome toll receptor cascade, red = Reactome innate immune system. All pathways were significantly altered (false discovery rate *q*-values < 0.01). (B) Table showing the core enriched genes for the two pathways. (C) Purified CD11b^+^Ly6C^+^CD115^high^ monocytes from PBS-treated (*n* = 6) or DT-treated (*n* = 6) treated mice were cultured overnight in medium with or without LPS. Graphs show the amount ± SEM of cytokines secreted into the culture supernatant. TNF-α PBS versus DT for LPS-treated cells, *p* = 0.022. Data are pooled from two independent experiments. Statistical analyses were carried out with a Student's unpaired *t*-test.

The effector function of Ly6C^+^ monocytes induced in the absence of DCs is likely to depend on the immune environment encountered at the time of expansion. Therefore, to understand the environmental cues to which DT-Ly6C^+^ monocytes might respond, we performed a gene set enrichment analysis with curated gene sets to compare the pathways of differentially expressed genes in monocytes from control or DC-depleted mice. DT-Ly6C^+^ monocytes were enriched for genes involved in TLR and innate signaling pathways compared with control cells, with several overlapping gene sets. The two highest-ranking pathways according to the normalized enrichment score are shown in Figure 6A. Figure 6B lists the genes that contribute to the leading-edge subset of the two pathways. In addition to genes specific for the response to LPS and TLR signaling pathways (*tlr* genes, *Ly86*, *CD180*, *Peli*), DT-Ly6C^+^ monocytes were also enriched for genes encoding proteins required for the production of cytokines involved in innate pro-inflammatory immune responses (*irf* genes, *Ikbk*, *Nfkb2*, *Zbp1*). Therefore, we hypothesized that Ly6C^+^ monocytes from DC-depleted mice would produce more inflammatory cytokines in response to LPS, than control cells. To test this hypothesis, we analyzed the production of several cytokines by sorted Ly6C^+^ monocytes from DC-depleted and DC-replete mice. Figure 6B shows that DT-Ly6C^+^ monocytes produced significantly more TNF-α than classical splenic Ly6C^+^ monocytes, while no significant differences were observed for the other cytokines tested. Together, these data support a model in which the depletion of DC leads to the accumulation of a differentiated population of monocytes that are poised for innate immune activation.

## Discussion

Over the last decade, the CD11c.DTR mice and other DC depletion models have provided a powerful tool to understand DC function 1,2,45, but the consequences of loss of DCs on other cells of the MPS have not been fully appreciated. In this study, we have characterized the splenic monocyte population that expands after the depletion of DCs from CD11c.DTR mice. We have shown that the loss of CD11c^+^ cells drives the accumulation of a variant population of CD64^+^Ly6C^+^ monocytes that upregulates the apparatus used for innate immune and TLR signaling.

Classical monocytes express high levels of Ly6C, and low levels of CD64 and MHC class II in the blood 19,33. Activation and/or extravasation into tissues results in the downregulation of Ly6C and upregulation of CD64 and MHC class II, resulting in a “cascade” of cells when these antigens are plotted on flow cytometric dot plots 20,33,40,46. This phenotypic transition as monocytes egress out of the vasculature is associated with the expression of a distinct tissue-dependent transcriptional signature (Fig. 2) 20. Here, we have identified a population of splenic monocytes that spontaneously express CD64, but that do not cluster with dermal monocytes in PCA. Therefore, the loss of DCs results in the expansion of a variant Ly6C^+^ splenic monocyte. However, DT-Ly6C^+^ cells also display characteristics associated with Ly6C^neg^ monocytes—for example, the upregulation of the transcription factor *Nr4a1* 47, and expression of TLRs and other molecules required for sensing of nucleic acids 5. Our findings therefore demonstrate that the distinction between individual monocyte populations may become blurred under conditions of acute depletion of CD11c^+^ myeloid cells when the cellular composition within the MPS is perturbed.

Recent publications have presented an emerging picture of control of homeostasis within the MPS by serum cytokines, notably M-CSF. Removal of populations that bind these cytokines will result in a decrease in the local concentration, which may impact on the proliferation and survival of other myeloid populations 10,48. DC populations act as a cellular sink for the growth factors G-CSF and Flt3(L) 25,26. We have shown that G-CSF is required for the expansion of splenic monocytes after the loss of DCs, and that exogenous G-CSF is sufficient to induce a variant monopoiesis that is similar to that seen in DC-depleted mice. Depletion of DCs also induces the expansion of splenic neutrophils that are functionally important for the clearance of bacterial infections in these models 26,30. Jiao et al. recent reported that the depletion of DCs from CD11c.DTR mice leads to neutrophilia in the spleen and tissues by enhancing recruitment out of the BM 31. We have shown that monocytes and neutrophils expand concurrently in the spleen after depletion of CD11c^+^ cells, and both populations expand in response to G-CSF. It is not possible however to infer causative mechanisms from these studies, and future work is needed to determine whether G-CSF has a direct or indirect effect on the expansion of monocytes in this model. Our data further demonstrate that cells that do not express CCR2 expand in the spleens of DT-treated recipients. While we cannot exclude a contribution from BM-derived cells, these data strongly support a role for extramedullary expansion of monocytes or their precursors. We did not detect differences in proliferation of Ly6C^+^ cells in the spleens of PBS- or DT-treated mice 48 h after treatment (data not shown), however it is possible that we missed the point of proliferation in our experiments. In addition, loss of CD11c^+^ cells may lead to local proliferation of splenic monocyte precursors, including cMoPs 9. Our findings are in line with previous data showing that constitutive ablation of DCs resulted in monocytosis due to a direct increase in hematopoiesis in the spleen 23.

Expression of CD11c is not restricted to DCs, and other cell types may be depleted from CD11c.DTR mice 29,49,50. We did not observe changes in lymphoid cell populations in our model, but F4/80^+^ macrophages were absent from the spleen 48 h after injection of DT. Removal of macrophages will lead to increases in serum M-CSF which could modulate monocyte populations 48. However, the depletion of DCs results in the expansion of monocytes and neutrophils in other mouse models in which DC populations are specifically perturbed, strongly suggesting that monocytosis in the CD11c.DTR mice is directly dependent on the ablation of DCs 27,31.

In conclusion we have shown that removal of CD11c^+^ cells from the MPS in the steady state is sufficient to drive the rapid transient expansion of a differentiated population of monocytes in the spleen, suggesting a model in which the loss of DCs drives the expansion of an effector monocyte population. Our kinetic analyses suggest a transient peak in the accumulation of splenic monocytes in DC-depleted mice. Therefore, there is a defined window in DT-injected mice in which DT-Ly6C^+^ monocytes may mediate responses to infectious or inflammatory challenge in the absence of DCs. These cells may be sensitive to the environment in which they emerge and, consistent with this concept, we have recently demonstrated that monocytes mobilized by injection of G-CSF produce iNOS, and become immunosuppressive, in a murine model of GVH disease 51.

It has been recently demonstrated that expression of the high-affinity DTR by DCs leads to hypocellularity in LN, but not in the spleen of CD11c.DTR mice 32. Together with our data, this study highlights the caution with which DC-depletion studies should be interpreted, particularly in the context of infection and/or inflammation.

## Materials and methods

### Mice

Animal protocols were approved by local institutional research committees and in accordance with U.K. Home Office guidelines. CD11c.DTR/GFP (B6.FVB-Tg(Itgax-DTR/ EGFP)57Lan/J) were used as both heterozygotes and homozygotes for the DTR/GFP transgene 52. *Ccr2*^−/−^ mice were a kind gift from Frederic Geissmann 39. CD11c^+^ DCs were depleted by a single i.p. injection of 100 ng of DT (Sigma, UK) in PBS 52,53. Depletion of DCs was confirmed in all experiments. To mobilize Ly6C^+^ monocytes C57BL/6 mice were injected s.c. for 5 days with rhuman G-CSF (200 μg/kg/day; Neupogen, Amgen, France) or PBS vehicle. G-CSF was neutralized by i.p. injection of two doses of 50 μg αG-CSF 24 h apart (Clone 67604, R&D Systems U.K.) adapted from published data [54]. Controls received either nonspecific IgG1 or PBS. The second dose was given immediately prior to the injection of PBS or DT.

### Competitive chimeras

Ten-week-old C57BL/6 mice received 11Gy X-ray irradiation before i.v. injection of a 1:1 mixture of CD11c.DTR.CD45.1.CD45.2 and CD11c.DTR.*Ccr2*^−/−^.CD45.2 BM cells (5 × 10^6^ total cells). Chimeric mice were used at ∼3 months post reconstitution.

### Flow cytometry

Spleens were digested with Collagenase IV (Worthington Biochemical Corporation). The following antibodies were used for flow cytometric analysis, all from eBiosciences or BD-Biosciences: FcBlock (clone 2.4G2), anti-CD11b (M1/70), anti-CD11c (HL3), anti-Ly-6C (AL-21), anti-Ly-6G (1A8), anti-CD45.1 (A20), anti-CD45.2 (104), anti-MHCII (M5/114.15.2), anti-CD115 (AFS98), anti-CD64, anti-F4/80 (BM8), anti-CD135 (A2F10), anti-CD117 (2B8). FSC-W and a lineage dump gate were used in some experiments to exclude irrelevant cells (anti-CD3 (145-2C11), anti-CD19 (1D3), anti-B220 (RA3-6B2), anti-NK-1.1 (PK136), and anti-Ly-6G (1A8)). Samples were acquired using a BD LSR Fortessa Cell Analyzer (BD Bioscience), and analyzed using FlowJo software (Treestar).

### Microscopy

Splenic CD11b^+^Ly6C^+^ cells were FACS-sorted and spun onto slides using a Cytospin 2 Centrifuge (Shandon Instruments). The cells were air-dried, fixed with methanol, and stained with Giemsa (Sigma Aldrich). Images were acquired using a Leica DMD108 (Leica Microsystems) microscope.

### Microarray analysis, normalization, and validation

Total RNA was extracted from sorted cells using the RNeasy Microkit (Qiagen, UK) and hybridized on Affymetrix Mogene 2.0 arrays at the UCL Cancer Institute core facility. A detailed description of the methods used is included in the Supporting Information.

### Luminex

Sorted monocytes were cultured for 12 h ± 100 ng/mL LPS (Sigma-Aldrich) at 2.5 × 10^5^ cells/mL in duplicate wells, and the supernatant analyzed with the FIDIS system using Luminex 100 software (LuminexCorp) and Mouse cytokine 10-plex panel (Life Technologies).

### Statistical analysis

Data were analyzed using Graphpad Prism 6 and statistical comparisons were made by using a two-tailed Student *t*-test for parametric data and a two-tailed Mann–Whitney *U* test for nonparametric data. The kinetic experiments were analyzed using a two-way ANOVA with multiple comparisons at different time points, and an adjusted p-value according to Sidak's multiple-comparison test.

## Abbreviations

cMoP: common monocyte progenitor
DT: diphtheria toxin
DTR: diphtheria toxin receptor
Flt3(L): fms-like tyrosine kinase receptor-3 (ligand)
MPS: mononuclear phagocyte system

## Supporting Information

As a service to our authors and readers, this journal provides supporting information supplied by the authors. Such materials are peer reviewed and may be re-organized for online delivery, but are not copy-edited or typeset. Technical support issues arising from supporting information (other than missing files) should be addressed to the authors.

Supporting Information.

Supplemental Figure 1. Depletion of CD11c+ cells in DT-injected CD11c.DTR mice. Supplemental Figure 2. Expansion of neutrophils in DT-treated recipients. Supplemental Figure 3. Monocytes in the dermis of DT-treated mice. Supplemental Figure 4. Gene profile comparisons of monocyte populations. Supplemental Figure 5. DT-Ly6C+ cells do not depend on the expansion of BM myeloid populations.
