# Supplementary material for: Depletion of CD11c+ cells in the CD11c.DTR model drives expansion of unique CD64+ Ly6C+ monocytes that are poised to release TNF-α
Source: Eur J Immunol. 2015 Nov 30;46(1):192–203. doi: 10.1002/eji.201545789 (PMC4722854; doi:10.1002/eji.201545789)
Supplement: Supplementary file 2 — Supplemental Figure 1. Depletion of CD11c+ cells in DT-injected CD11c.DTR mice. Supplemental Figure 2. Expansion of neutrophils in DT-treated recipients. Supplemental Figure 3. Monocytes in the dermis of DT-treated mice. Supplemental Figure 4. Gene profile comparisons of monocyte populations. Supplemental Figure 5. DT-Ly6C+ cells do not depend on the expansion of BM myeloid populations. [file eji0046-0192-sd2.pdf]

European Journal of Immunology

Supporting Information

for

DOI 10.1002/eji.201545789

Shivajanani Sivakumaran, Stephen Henderson, Sophie Ward, Pedro Santos E. Sousa, Teresa Manzo, Lei Zhang, Thomas Conlan, Terry K. Means, Maud D'Aveni, Olivier Hermine, Marie-Thérèse Rubio, Ronjon Chakraverty and Clare L. Bennett

Depletion of CD11c^{+} cells in the CD11c.DTR model drives expansion of unique CD64^{+} Ly6C^{+} monocytes that are poised to release TNF-α

© 2015 WILEY-VCH Verlag GmbH & Co. KGaA, Weinheim

Depletion of CD11c$^+$ cells in the CD11c-DTR model drives expansion of unique CD64$^+$ Ly6C$^+$ monocytes that are poised to release TNFα.

Supporting methods:

Microarray Analysis, Normalization and Validation.

To analyze the data from microarray experiments UCL Affymetrix Mogene 2.0 and Immgen Affymetrix Mogene 1.0 were background corrected, preprocessed and normalized separately using the "oligo" package from Bioconductor [1], specifically the "rma" method [2]. Expression data was then filtered to contain only probe sets present on both Mogene 1.0 and 2.0 platforms. They were combined and renormalized to remove batch effects using the "sva" package from Bioconductor ("ComBat" method) [3]. To show the relationship between sample groups we used the R programming language to plot principal components and classic multidimensional scaling projections of the samples. Significantly differentially expressed genes were selected using the limma method from Bioconductor [4]. The Neutrophil expression data used for Figure 4 were quantile normalized to the PBS Ly6C$^+$ and DT Ly6C$^+$ samples for comparison of relative gene expression.

We used gene set enrichment analysis [5] to determine expression overlap between our expression data and other curated signatures in the Broad Institute's online database: MsigDB [6].

The microarray data are available in the Gene Expression Omnibus (GEO) database (http://www.ncbi.nlm.nih.gov/gds; GSE58263). Data published by Tamoutounour *et al* is available under accession numbers GSM1198073-110 [7].

Supplemental Figure 1. Depletion of $CD11c^{+}$ cells in DT-injected CD11c.DTR mice.

A. CD11c.DTR mice were injected with PBS or DT and splenocytes analyzed 48 hours later. *Left* - Representative dot plots show the depletion of $CD11c^{+}GFP^{+}$ cells from the spleens of CD11c.DTR mice after a single injection of DT. *Right* – Graphs show the frequency and number ±SEM of $CD11c^{+}GFP^{+}$ cells in the spleen of PBS- (n=61) and DT- (n=75) mice. Frequency of $CD11c^{+}GFP^{+}$ cells *P*<.0001; number of $CD11c^{+}GFP^{+}$ cells *P*<.0001. Data are pooled from multiple experiments. **B.** Graph shows the frequency ±SEM of $CD11b^{+}Ly6C^{low}F4/80^{+}$ cells in the spleen of PBS- (n=10) and DT- (n=8) mice, *P*<.0001. Data are pooled from 2 independent experiments. Statistical analyses were carried out with a Mann Whitney test. **C.** Graphs showing the percentage ±SEM of gated lymphoid cells in the spleen of PBS- or DT-treated mice. Data are pooled from 3 independent experiments.

Supplemental Figure 2. Expansion of neutrophils in DT-treated recipients.

CD11c.DTR mice were injected with PBS or DT and analyzed 48 hours later. **A.** *Left* - Graph shows the percentage ±SEM of $CD11b^{+}Ly6C^{int}$ neutrophils in the spleen of PBS- (white) and DTR- (grey) treated recipients at different times post-

injection of DT. *Right* - Graph shows the number ±SEM of CD11b$^+$Ly6C^{int} neutrophils. 2-way ANOVA with multiple comparisons (*) PBS versus DT: frequency day 1 P=.0355, day 2 P<.0001, day 4 P=.0357; number day 2 P<.0001. Data are pooled from multiple independent experiments: day 1 PBS/DT n=6; day 2 PBS/DT n=26/24; day 4 PBS/DT n=6. **B.** *Left* - Graph showing the frequency ±SEM of neutrophils in the blood and BM of PBS- (white triangles and squares) and DT- (grey triangles and squares) treated recipients. 2-way ANOVA with multiple comparisons (*) day 1 blood P=.0005, day 2 BM and blood P=.0020 and P=.0440 respectively, day 4 blood P=.0090. *Right* - Graph showing the number ±SEM of neutrophils in the BM from the femurs and tibias of one back leg of PBS- and DT- treated recipients. There is no significant difference between the number of cells in the two groups. Data are pooled from multiple independent experiments, days 1 and 4 PBS/DT n=6, day 2 BM PBS/DT n=14/12, blood PBS/DT n=12. **C.** Graph showing the percentage of Ly6C$^+$ or Ly6G$^+$ splenocytes of live cells in PBS- or DT-treated mice (n=9 per group). Cells are gated from dot plots equivalent to that show in Figure 1A. Ly6C$^+$ cells PBS versus DT P=.0030, Ly6G$^+$ cells; Mann-Whitney test (*) P=.0142. Data are pooled from 3 independent experiments. **D.** Langerin-DTR mice were injected with PBS or DT and analyzed 48 hours later. Representative dot plots show the frequency of cells expressing Ly6C and CD11b in the spleen (*left*) and skin epidermis (*right*). *Far right* - dot plots show depletion of CD11b$^+$CD11c$^+$ Langerhans cells from the epidermis of DT- but not PBS-treated mice. Data are representative of 2 mice from 1 experiment.

Supplemental Figure 3. Monocytes in the dermis of DT-treated mice.

A. Graph showing the frequency ±SEM of $CD11b^{+}Ly6C^{high}$ cells of $lineage^{neg}$ cells in the dermis of PBS- or DT-treated CD11c.DTR mice 48 hours after injection. PBS n = 9, DT n= 8; data are pooled from 3 independent experiments. **B.** *Left* - representative contour plots showing $Ly6C^{high}$ splenocytes and dermal cells in DT-treated mice. *Right* - histograms showing the median fluorescent intensity of CD64 on cells from gated $Ly6C^{high}$ populations in the spleen and dermis of DT-treated recipients. Data are representative from 8-9 mice in 3 independent experiments.

Supplemental Figure 4. Gene profile comparisons of monocyte populations.

Principal Components Analysis (PCA) of gene expression by dermal monocyte/macrophage subsets published by Tamoutounour *et al* [7]. The most variability between these cells occurred along PC1.

Supplemental Figure 5. DT-$Ly6C^{+}$ cells do not depend on the expansion of BM myeloid populations.

A. *Left* - Representative flow cytometry plots showing gating strategy used to identify MDP (macrophage/DC precursors) and cMoP (common monocyte progenitors) in the BM of PBS- and DT-injected mice. Cells were pre-gated on $lineage^{neg}CD115^{+}$ cells according to the protocol by Hettinger *et al* [8]. *Right* –

Graph showing the frequency ±SEM of progenitor and monocyte populations in the BM of PBS- (n=11) and DT- (n=9) mice. Ly6C^{+} monocytes PBS vs DT *P*<.0001. Data are pooled from 3 independent experiments. Statistical analyses were carried out with a Student's unpaired T-test. **B.** Graph showing the percentage ±SEM of CD11b^{+}Ly6C^{+}CD115^{high} monocytes in the blood of *Ccr2*$^{-/-}$ .CD11c.DTR mice 48 hours after injection of PBS (n=4) or DT (n=4). Data are pooled from 3 independent experiments.

Sivakumaran et al Supplemental Figure 1.

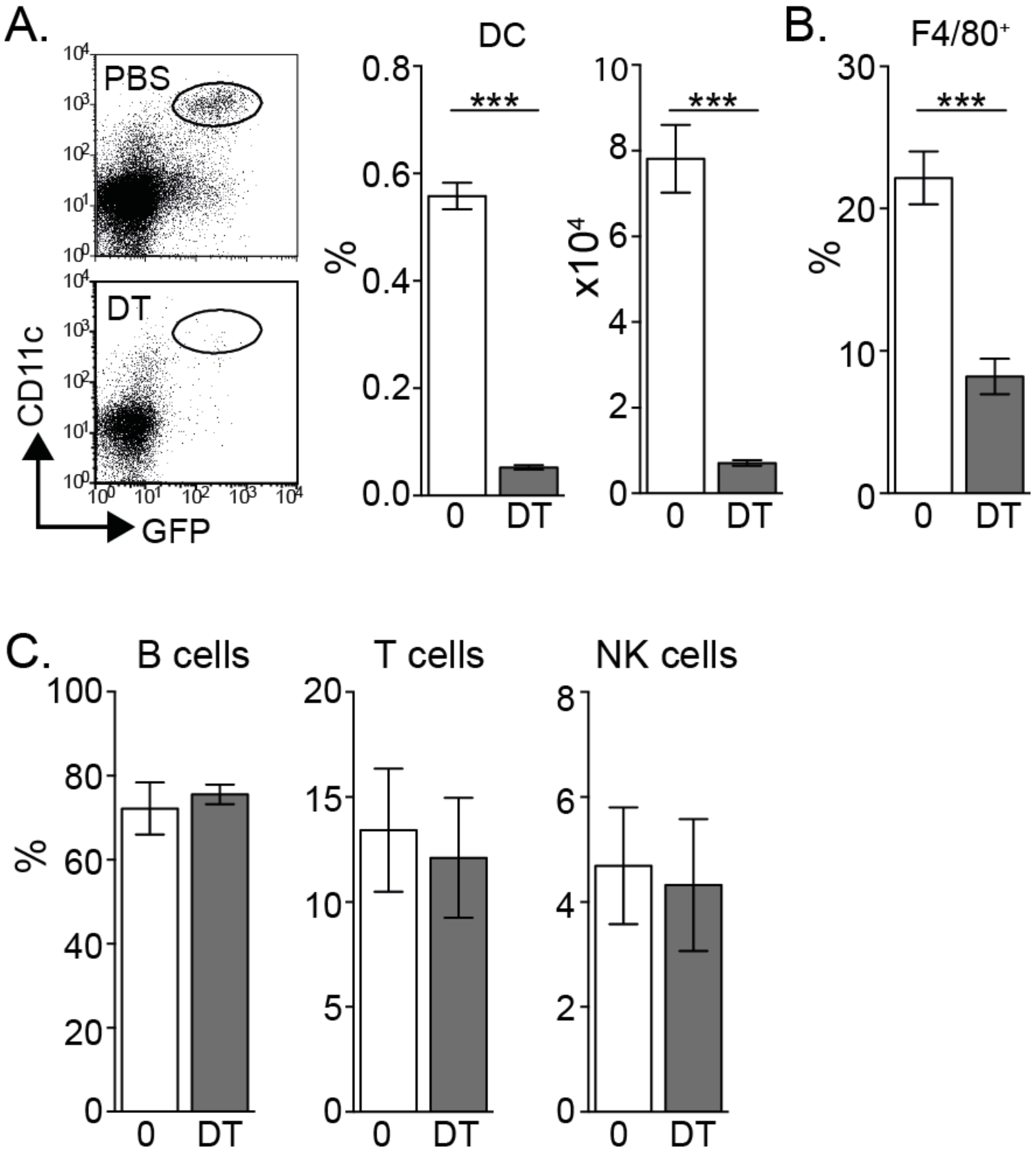

Sivakumaran et al Supplemental Figure 2.

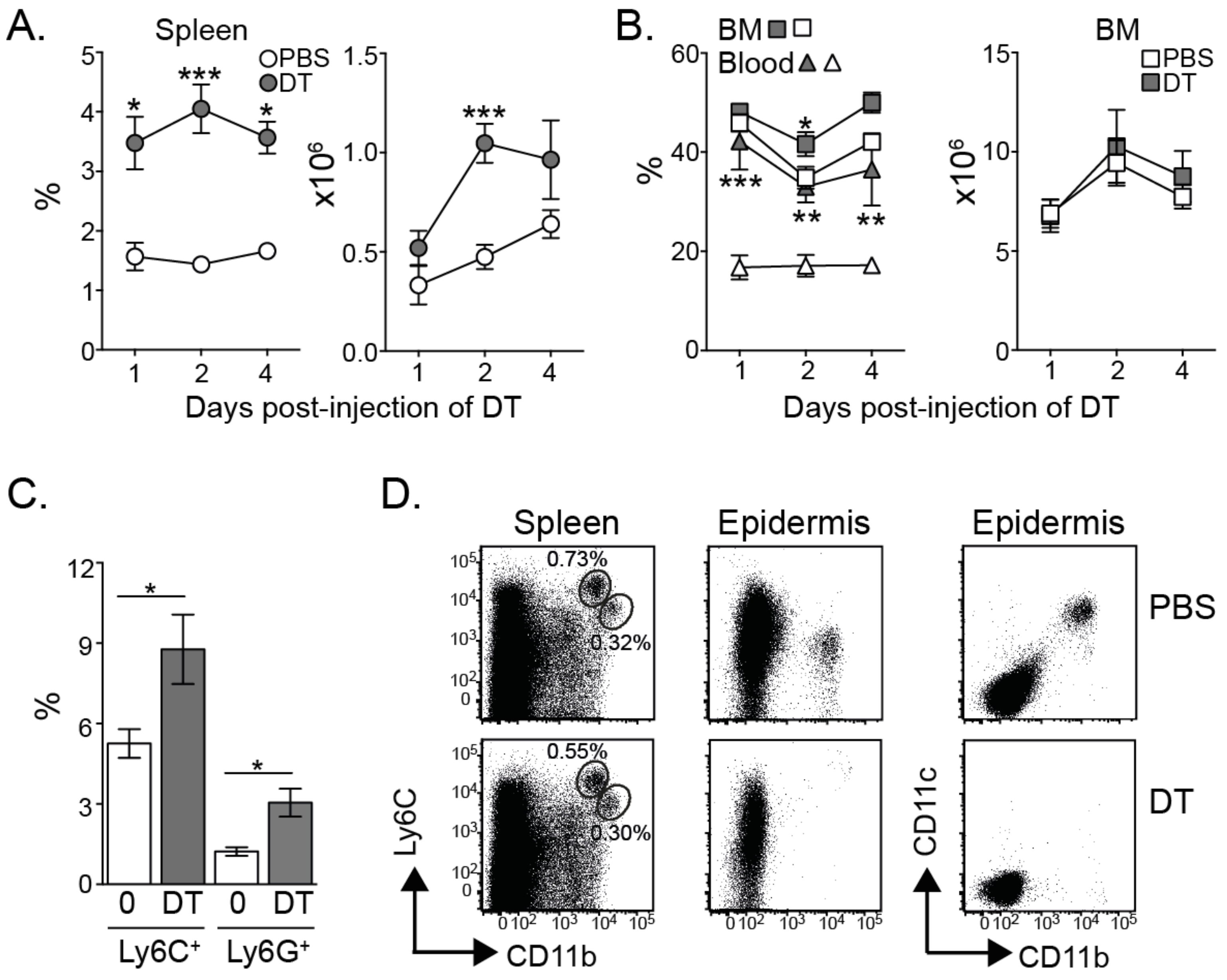

Sivakumaran et al Supplemental Figure 3.

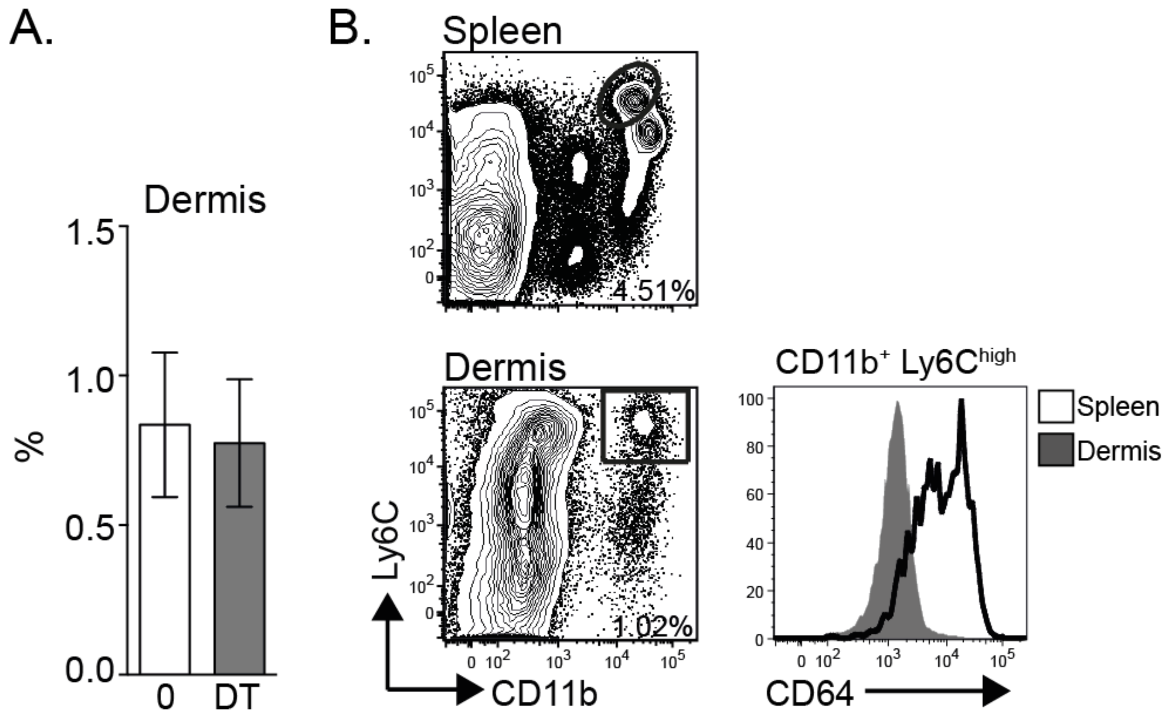

Sivakumaran et al Supplemental Figure 4.

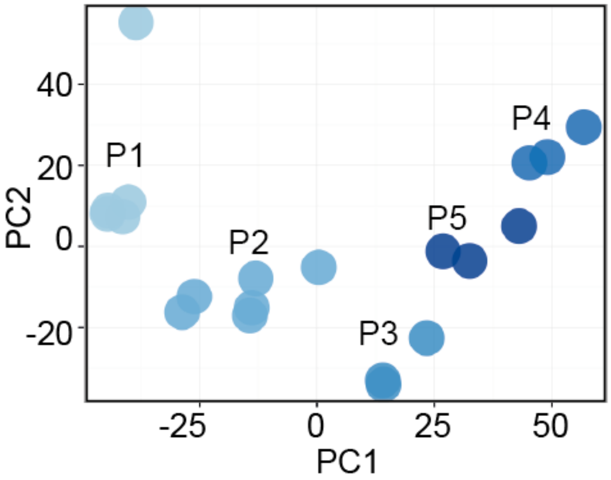

Sivakumaran et al Supplemental Figure 5.

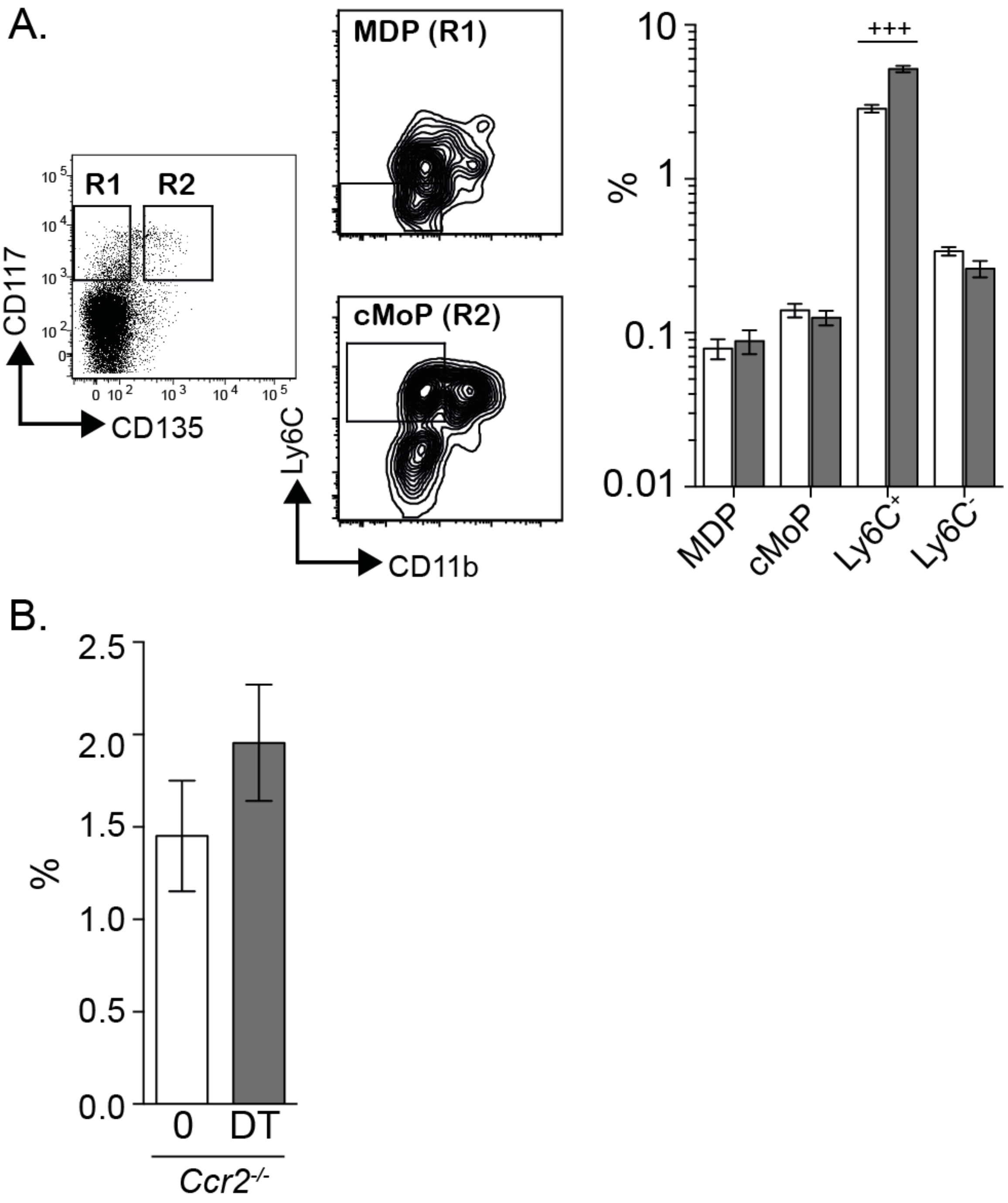

References

1 **Gautier, L., Cope, L., Bolstad, B. M. and Irizarry, R. A.,** affy--analysis of Affymetrix GeneChip data at the probe level. *Bioinformatics* 2004. **20**: 307-315.

2 **Carvalho, B. S. and Irizarry, R. A.,** A framework for oligonucleotide microarray preprocessing. *Bioinformatics* 2010. **26**: 2363-2367.

3 **Leek, J. T., Johnson, W. E., Parker, H. S., Jaffe, A. E. and Storey, J. D.,** The sva package for removing batch effects and other unwanted variation in high-throughput experiments. *Bioinformatics* 2012. **28**: 882-883.

4 **Smyth, G. K.,** Limma: linear models for microarray data. In **R. Gentleman, V. C., S. Dudoit, R. Irizarry, W. Huber** (Ed.) *Bioinformatics and Computational Biology Solutions using R and Bioconductor*. Springer, New York 2005, pp 397-420.

5 **Smyth, G. K., Michaud, J. and Scott, H. S.,** Use of within-array replicate spots for assessing differential expression in microarray experiments. *Bioinformatics* 2005. **21**: 2067-2075.

6 **Subramanian, A., Tamayo, P., Mootha, V. K., Mukherjee, S., Ebert, B. L., Gillette, M. A., Paulovich, A., Pomeroy, S. L., Golub, T. R., Lander, E. S. and Mesirov, J. P.,** Gene set enrichment analysis: a knowledge-based approach for interpreting genome-wide expression profiles. *Proceedings of the National Academy of Sciences of the United States of America* 2005. **102**: 15545-15550.

7 **Tamoutounour, S., Guilliams, M., Montanana Sanchis, F., Liu, H., Terhorst, D., Malosse, C., Pollet, E., Ardouin, L., Luche, H., Sanchez, C., Dalod, M., Malissen, B. and Henri, S.,** Origins and functional specialization of macrophages and of conventional and monocyte-derived dendritic cells in mouse skin. *Immunity* 2013. **39**: 925-938.

8 **Hettinger, J., Richards, D. M., Hansson, J., Barra, M. M., Joschko, A. C., Krijgsveld, J. and Feuerer, M.,** Origin of monocytes and macrophages in a committed progenitor. *Nature immunology* 2013. **14**: 821-830.
