# Supplementary material for: Depletion of CD11c+ cells in the CD11c.DTR model drives expansion of unique CD64+ Ly6C+ monocytes that are poised to release TNF-α
Source: Eur J Immunol. 2015 Nov 30;46(1):192–203. doi: 10.1002/eji.201545789 (PMC4722854; doi:10.1002/eji.201545789)
Supplement: Supplementary file 1 — Supporting Information. [file eji0046-0192-sd1.pdf]

European Journal of Immunology

Peer review correspondence

Depletion of CD11c^{+} cells in the CD11c.DTR model drives expansion of unique CD64^{+} Ly6C^{+} monocytes that are poised to release TNF-α

Shivajanani Sivakumaran, Stephen Henderson, Sophie Ward, Pedro Santos E Sousa, Teresa Manzo, Lei Zhang, Thomas Conlan, Terry K. Means, Maud D'Aveni, Olivier Hermine, Marie-Thérèse Rubio, Ronjon Chakraverty and Clare L. Bennett

Corresponding author: Clare L. Bennett, Department of Haematology, UCL Royal Free Campus, Rowland Hill Street, London, United Kingdom

Review Timeline:	Submission date:	7 May 2015
	First editorial decision:	27 May 2015
	First revision received:	20 August 2015
	Second editorial decision:	8 September 2015
	Second revision received:	10 September 2015
	Accepted:	7 October 2015

Handling Executive Committee members: Prof. Bernard Malissen

Please note that the correspondence below does not include the standard editorial instructions regarding preparation and submission of revised manuscripts, only the scientific revisions requested and addressed.

First Editorial Decision – 27 May 2015

Dear Dr. Bennett,

Manuscript ID eji.201545789 entitled "Depletion of dendritic cells in the CD11c-DTR model drives expansion of unique CD64+ Ly6C+ monocytes that are poised to release TNFα" which you submitted to the European Journal of Immunology has been reviewed.

The comments of the referees are included at the bottom of this letter. A revised version of your manuscript that takes into account the comments of the referees will be reconsidered for publication. Should you disagree with any of the referees' concerns, you should address this in your point-by-point response and provide solid scientific reasons for why you will not make the requested changes.

You should also pay close attention to the editorial comments included below. **In particular, please edit your figure legends to follow Journal standards as outlined in the editorial comments. Failure to do this will result in delays in the re-review process.**

Please note that submitting a revision of your manuscript does not guarantee eventual acceptance, and that your revision will be re-reviewed by the referees before a decision is rendered.

If the revision of the paper is expected to take more than three months, please inform the editorial office. Revisions taking longer than six months may be assessed by new referee(s) to ensure the relevance and timeliness of the data.

Once again, thank you for submitting your manuscript to European Journal of Immunology and we look forward to receiving your revision.

Yours sincerely,
Laura Soto Vazquez

On behalf of Prof. Bernard Malissen

Editorial Office
European Journal of Immunology
e-mail: ejied@wiley.com
www.eji-journal.eu

****

Reviewer: 1

Comments to the Author
In this paper, Sivakumaran et al explore the monocyte population which expands in the spleen after depletion of CD11c+ cells using the DTR system. They show there is accumulation of a population of Ly6C+MHCII+ cells which have transcriptional similarities to classical Ly6Chi monocytes, but which are distinct by being more differentiated and having pro-inflammatory potential. The expansion of these monocytes is independent of CCR2, but reflects an indirect effect of G-CSF. The data presented are fairly clear, although they are not entirely novel, given that it is well known that depletion of CD11c+ cells in vivo leads to an inflammatory disorder associated with the pro-inflammatory myeloid cells such as monocytes and neutrophils which are shown to accumulate here.

European Journal of Immunology

Furthermore, the study raises many unanswered questions which warrant attention:

1) While it may not alter the data shown here, it needs to be acknowledged that it is now clear that CD11c-cre based models including the DTR system are not specific to "DC". Several studies have shown that this can also affect other cell lineages such as T cells, plasma cells and most importantly, macrophages. This latter case is particularly relevant when considering the reasons behind the secondary expansion of monocytes, with possible roles for eg loss of CSF-1R+ cells, deficiencies in myeloid lineage cytokines etc. That the authors' results may not reflect depletion of genuine DC could be consistent with the absence of monocyte expansion in langerin-DTR mice, where there will be loss of mature langerin+ DC.

2) The authors do not present direct evidence that the Ly6C+MHCII+ population is absent from the steady state spleen, but simply show that the total population of Ly6C+ monocytes upregulate MHCII, F4/80 etc after depletion of CD11c+ cells. As several studies (including those by Tamoutounour et al and Jakubzick et al cited here) have shown the presence of MHCII+ monocytes in steady state skin, LN, intestine and lung, it should be shown whether these are also be detectable in the spleen.

3) In respect of this subset of monocytes, it should also be noted that most evidence (including Ref#19) indicates that these are derived from Ly6C+MHCII- monocytes in the bloodstream, rather than circulating Ly6C+MHCII+ monocytes as suggested here.

4) For these reasons, it would be important to know how Ly6ChiMHCII- monocyte numbers behave in the bloodstream and spleen after CD11c+ cell depletion. Blood Ly6ChiMHCII- monocytes should also have been included in the microarray experiments.

5) It would be important to know whether the expansion of neutrophils seen in the spleen or elsewhere of CD11c cell depleted mice precedes that of monocytes. Not only might that help understand how G-CSF might be relevant to the monocyte expansion, but it would also indicate whether an initial inflammatory reaction might trigger the monocyte accumulation.

6) For this purpose, Ly6G should be used throughout the study to identify neutrophils directly.

7) The data on CCR2 are interesting, but require further detail to allow interpretation. For instance, does the CCR2-independent monocyte accumulation occur in other tissues where it is known that this mechanism is needed for monocyte entry in steady state and/or inflammation, such as the intestine, lung, heart, skin? If restricted to the spleen, does this reflect in situ proliferation of pre-existing precursors or Ly6C+MHCII+ (or Ly6C+MHCII-) monocytes. BrdU incorporation studies combined with the additional phenotyping indicated above would allow this to be determined.

8) The apparently indirect effect G-CSF is also interesting, but again, its significance and underlying mechanism have not been explored. As noted above, does this suggest that eg neutrophils are needed to produce monocytopoietic factors such as CSF-1, or non-CCR2 dependent chemokines?

9) In this respect, it would have been interesting to examine the role of CSF-1 in the monocyte accumulation?

10) The gene expression patterns used to conclude that this is a "discrete" population of pro-inflammatory monocytes are rather selective, a reservation underlined by the fact that a modest increase in TNF production is the only functional difference between DTR treated and control spleen monocytes. Most

European Journal of Immunology

monocytes are "poised" for innate inflammatory responses and the majority of the genes which are upregulated are features of differentiation within the monocyte/macrophage lineage. Thus it would be important to know eg how the genes shown in Figure 6B are expressed in dermal macrophages. Even if these do not show complete overlap, it is quite possible that any differences reflect tissue-determined variation rather than "inflammation".

11) The authors do not really discuss what the wider implications of their results are for understanding immune function.

Reviewer: 2

Comments to the Author

The study by Sivakumaran et al. demonstrates that diphtheria toxin mediated depletion of CD11c+ cells in CD11c-DTR mice leads to a major change in phenotype of Ly-6C+ monocytes in the spleen. These findings are potentially highly relevant to the interpretation of the wealth of studies that have used and continue to use CD11c-DTR mice to ascribe function to dendritic cells (and/or CD11c+ macrophages).

While the authors describe aspects of the mechanism leading to generation of ‘variant’ splenic monocytes (eg a role for G-CSF), the underlying cause remains unclear. In this respect, it is important that the authors acknowledge the range of cells directly depleted by DT in these animals (ie. CD11c+ macrophages as well as dendritic cells) that could impact splenic monocyte phenotype. Furthermore, it needs to be determining whether the variant phenotype is restricted to the spleen or is a feature shared by the expanded populations of blood Ly-6C+ monocytes, to understand if this is a splenic micro-environment issue, verify the relationship of expanded blood monocytes to those in the spleen, and ascertain whether this study has broader implications when using CD11c-DTR mice to study other tissues (see Major concern 1). I have a number of additional concerns about data interpretation and controls required to ensure validity of the conclusions.

Major concerns:

1. Although the CD11c-DTR mouse was designed to specifically ablate conventional DC, it is clear from numerous publications that administration of DT in this system results in ablation of many tissue macrophages (Rivollier et al. 2012 JEM, Bain et al. 2013 Mucosal Immunol., Landsman et al. 2007 JI). The authors should acknowledge the potential impact of macrophage ablation on the appearance of these ‘variant’ Ly6C+ monocytes. This is not a trivial point as the authors state that the premise of the study is to interrogate “the impact of DC depletion on other cells of the immune system”. In fact, splenic macrophages are known to express CD11c to some degree. Therefore the CD64+ variant monocytes could be cells part-way differentiating to replenishing this population. The authors should include data on

the effect of DT administration on F4/80+ CD11b+ splenic macrophages, and of need, the title changed accordingly. (Indeed, Supp. Fig. 1 appears to also show loss of a GFP+ CD11bdull splenic population upon DT treatment – the authors should identify this GFP+ CD11bdull splenic population). The authors should also show if the expanded blood monocytes express the variant phenotype.

2. In Figure 1, the authors should show much more complete gating of different myeloid cells in blood and spleen – this could be Supplementary Figure 1. The authors should show Ly6G expression by the SSCint Ly6C+ CD115int cells to confirm their designation as neutrophils.

3. In Figure 2, in order to be able to assess whether these DT-elicited Ly6C+ monocytes express the markers shown in part B, an isotype control needs to be included. From the data shown, it is unclear whether there is any meaningful expression of CD64 by the DT-elicited monocytes. Similarly, CD11c expression by these monocytes appears negligible. Given that CD64 expression is in the title of the manuscript, this must be addressed.

4. According the text, the axes in Figure 4 are labelled incorrectly. Also, it is unclear what the graph in 4B is showing. According to the legend, it shows “the frequency of WT or Ccr2–/– cells within the CD11b+ Ly6C+ monocyte population”. If this is correct, what is making up the other 99% of cells? Part C is said to represent numbers but the axis is %.

5. In Figure 5, treatment with exogenous G-CSF is purported to ‘expand’ and ‘mobilize’ a population of Ly6C+ monocytes that are transcriptionally indistinguishable from DT-Ly6C+ monocytes. The transcriptomic data presented supports the conclusion that G-CSF causes ‘differentiation’ of monocytes to the DT-Ly6C+ phenotype, but no data is provided that shows the number of monocytes is ‘expanded’ or monocytes are ‘mobilized’ – please show the data for numbers in the blood and spleen or change the text accordingly.

6. In Figure 7 the authors test the production of cytokines in response to LPS stimulation. They show TNFα and IL6 and state “no significant differences were observed for the other cytokines”. The authors should indicate some of the other cytokines measured. Analysis of the kinetics of the appearance, persistence and contraction of DT-elicited Ly6C+ monocytes (Point 4) will allow the reader to assess the functional consequences of the presence of monocytes with heightened responsiveness to TLR stimulation.

7. In the Discussion, the authors claim to have identified a population of monocytes in spleen that “spontaneously express proteins associated with entry into tissues” – this reviewer is not entirely clear what markers the authors are referring to. Ly6Chi monocytes arriving in the dermis of the skin or in the intestinal mucosa begin to downregulate Ly6C and CCR2, and upregulate MHCII – DT-elicited Ly6C+ monocytes have high levels of Ly6C and CCR2, and fail to express MHCII. Therefore the phenotypic similarity between DT-elicited cells and recently extravasated monocytes in tissues is limited. Because the authors place such emphasis on CD64 expression, it would be informative if they compared the expression levels of CD64 on DT-Ly6C+ monocytes with monocytes in the dermis, or at least a representative tissue macrophage to allow one to establish the extent of CD64 expression.

Minor concerns:

1. In the Introduction the authors state that ‘Classical’ or ‘inflammatory’ Ly6C+ monocytes are released from the BM in response to infection via the ligation of the chemokine receptor CCR2”. This sentence is misleading as it suggests that these monocytes are only elicited by infection, whereas it is clear that these monocytes are released continuously into the blood of unmanipulated mice.

2. On page 9, the authors state “The heat map in Figure 3B shows an incremental increase in the expression level of most of the genes tested in the progression to DT-Ly6C+ monocytes” – this is unclear.

Reviewer: 3

Comments to the Author

In the paper entitled “ Depletion of dendritic cells in the CD11c-DTR model drives expansion of unique CD64+ Ly6C+ monocytes that are poised to release TNFα, Sivakumaran and colleagues have attempted to better characterize the monocyte influx observed upon DT injection on CD11c-DTR mice.

As shown on figure 1 and as previously published, the authors observed an increase in frequency of monocytes as well as neutrophils 48h after injection of DT in CD11c-DTR mice.

As the authors mainly focused on the spleen, and considering that in the original paper describing the CD11cDTR mice (Jung et al, Immunity, 2002) it was mentioned that some T cells may express CD11c and thus might be depleted upon DT treatment, the authors should maybe provide an overall study of the consequences of DT treatment on all cell types within the spleen (B cells, CD4+ T cells, CD8+ T cells, DC, monocytes, neutrophils, macrophages).

This influx of monocytes and neutrophils could be seen as a sign of inflammation.

As it is not observed with Langerin-DTR mice (by the way those results should be shown) in which the cell depletion within the spleen might be too subtle to trigger any inflammatory call, it would be of great interest to study if other DTR mouse model with significant cell depletion within the spleen might induce similar monocyte and neutrophil recruitment or if this phenotype is uniquely particular to the CD11c-DTR mouse model.

As presented on figure 1, less than 30 h post DT treatment, the percentage of monocytes in the spleen are significantly increased whereas the absolute numbers are unchanged. Nevertheless, 24h later this increase is significant for the frequency as well as the absolute numbers.

48h post DT treatment, the monocyte frequency within the BM is significantly increased whereas the absolute numbers are unchanged. Did the authors checked if later on the absolute numbers within the BM might be significantly increased as it happened with the spleen? Could the authors discuss this issue ?

European Journal of Immunology

A presented on figure 2, those monocytes appeared to have fragmented nucleus and slightly more vacuoles and they slightly increased their expression of CD64 and F4/80 which are typical macrophage markers. Interestingly, those monocytes do not upregulate CD11c and MHCII. Altogether, the phenotype changes appeared quite subtle. It would be interesting to also complete with the analysis of neutrophils (morphology and surface-marker phenotype) as they are also increased in numbers and later on in the paper the authors will assess if those cells could be related to neutrophils. Moreover, it would be interesting to compare side by side CD64 and F4/80 expression by DT-Ly6C+ as well as tissue macrophages and inflammatory monocytes observed in Listeria or toxoplasma infected mice.

According to Figure 3D, DT Ly6C+ monocytes clustered with PBS Ly6C+ monocytes and are also closely related to Dermis-P1 and dermis-P2 which are highly related to CCR2-dependent blood monocytes
Thus the use of competitive BM chimera combining WT and Ccr2-/- bone marrow was absolutely required in this study. Unfortunately, the authors do not seem to exploit properly the results. First of all, the Ccr2-/- are supposedly CD45.2+ and thus the WT should be followed as CD45.2+CD45.1+ and the Ccr2-/- should be CD45.2+ whereas on the figure 4, the Ccr2-/- are followed as CD45.1+ CD45.2-. The monocytes analysed in PBS should be considered as conventional monocyte and thus CCR2 dependent. Thus in the chimera mice, the ratio of 71.8% versus 27.2% observed on Figure 4B should be interpreted as the ratio of CCR2 dependent monocyte and should also be the ratio observed for blood monocyte whereas other cells within the blood such as B cells or neutrophils should have a 50-50 ratio. As a consequence, the ratio observed in DT treated mice (71.2% versus 27.7%) should be seen as CCR2-dependency of those DT Ly6C+ cells. Moreover figure 4C should be taken carefully as Ccr2-/- mice have definitely some leakiness and still have some monocytes within the periphery. The ratio no DT /DT seems to be similar between WT and Ccr2-/- mice. Thus those DT Ly6C+ cells might definitely be considered as Ccr2 dependent and thus of monocyte origin.

Regarding the data presented on Figure5, it would be important to include the control consisting of PBS + αG-CSF to compare with DT + αG-CSF.
The comparison with the neutrophils presented on Figure 5D should be done using neutrophils isolated from DT treated spleens.

The comparison presented on Figure 5C should be completed with splenic monocytes isolated from listeria or toxoplasma infected mice as those cells might be related to the inflammatory monocytes known to produce TNF as presented on figure 6.

At the end of this paper, I am not sure to be completely convinced that those DT Ly6Chigh cells are not related to inflammatory monocytes. The authors mentioned in the discussion that they display many characteristics associated with Ly6Cneg monocytes but they did not really compared their signature.

European Journal of Immunology

It is not really clear to me neither if those cells are expanding extramedullary (Fig 1D) and independently of CCR2.

Finally, the authors did not really prove that those iNOS producing monocytes were immunospressive and they did not provide any proof that any conclusions made with the CD11cDTR mice upon DT treatment was not likely due to the depletion of DC but rather to the presence of those Ly6C+ cells in the spleen or even to the neutrophils.

Minor points:

Significant number of mistakes within the figure legends:

Fig1A: it is starting by “left” whereas right and left are concerned

Fig1B right and left are inverted; It is repeatedly written DTR-treated recipients instead of DT-treated recipient

Fig1D Why not providing the exact number of mouse used to calculate the statistics instead of n=14-19 and n=11-16

Fig2A Geisma instead of Giemsa

Fig2C: why did the authors use different gating strategy to follow the potentially similar (or not) cells: CD11b+Ly6Chigh or CD11b+Ly6ChighCD115high

Fig4B: not only the axe labels might be wrong (CD45.1 / CD45.2) but also the plot representing the chimerism gives a percentage with values below 0.8. Can the frequency be below 0.8% or the authors meant 80 % ?

Fig4C: the legend says that the graph shows the numbers of CD11b+Ly6C+ whereas on the figure itself it is labeled as percentage.

Instead of mentioning “statistical analyses were carried out with a Student’s unpaired T-test or Mann Whitney as appropriate, could the authors specify which statistical test they used for each plot. This is an important information for the reader.

<u>First revision – authors’ response – 20 August 2015</u>

Reviewer: 1

Comments to the Author

In this paper, Sivakumaran et al explore the monocyte population which expands in the spleen after depletion of CD11c+ cells using the DTR system. They show there is accumulation of a population of Ly6C+MHCII+ cells which have transcriptional similarities to classical Ly6Chi monocytes, but which are distinct by being more differentiated and having pro-inflammatory potential. The expansion of these monocytes is independent of CCR2, but reflects an indirect effect of G-CSF. The data presented are fairly clear, although they are not entirely novel, given that it is well known that depletion of CD11c+ cells in vivo

European Journal of Immunology

leads to an inflammatory disorder associated with the pro-inflammatory myeloid cells such as monocytes and neutrophils which are shown to accumulate here. Furthermore, the study raises many unanswered questions which warrant attention:

1) While it may not alter the data shown here, it needs to be acknowledged that it is now clear that CD11c-cre based models including the DTR system are not specific to "DC". Several studies have shown that this can also affect other cell lineages such as T cells, plasma cells and most importantly, macrophages. This latter case is particularly relevant when considering the reasons behind the secondary expansion of monocytes, with possible roles for eg loss of CSF-1R+ cells, deficiencies in myeloid lineage cytokines etc. That the authors' results may not reflect depletion of genuine DC could be consistent with the absence of monocyte expansion in langerin-DTR mice, where there will be loss of mature langerin+ DC.

As the reviewer points out, the CD11c promoter used by Steffen Jung to control expression of the high affinity DTR in the CD11c-DTR mice has been shown to function in other cell types, principally some macrophage populations and activated T cells (Probst *et al* (2005) Clin Exp Immunol 141: 398; Zammit *et al* (2002) Immunity 22: 561). We have characterized depletion of other cell types in our model and have shown that F4/80+ macrophages are depleted from the spleen of the CD11c-DTR mice, but other immune cell populations are not affected. These data have now been included in Supplemental Figure 1 of the manuscript.

We agree that loss of macrophage may also result in the environmental changes that lead to expansion of monocytes. However, monocytosis has also been demonstrated on depletion of DC from the zDC-DTR mice in which the DTR is expressed from a promoter for the transcription factor *zbtb46*. This protein is highly expressed in DC lineage populations but not in macrophages or other myeloid cells (Meredith *et al* (2012) J Exp Med 209: 1153)). In addition, the neutrophilia, which accompanies monocytosis in this model, has recently been shown to occur in a number of murine models that lack normal DC populations (e.g. Flt3L-/- mice, Batf3-/-; Jiao *et al* (2014) J Immunol 192: 3374). For these reasons we believe that the expansion of monocytes and neutrophils upon injection of DT into CD11c-DTR mice is due to depletion of DC and not other myeloid populations. We have included a discussion of this point in our manuscript (page 8 paragraph 1 and page 18 paragraph 1).

2) The authors do not present direct evidence that the Ly6C+MHCII+ population is absent from the steady state spleen, but simply show that the total population of Ly6C+ monocytes upregulate MHCII, F4/80 etc after depletion of CD11c+ cells. As several studies (including those by Tamoutounour et al and Jakubzick et al cited here) have shown the presence of MHCII+ monocytes in steady state skin, LN, intestine and lung, it should be shown whether these are also be detectable in the spleen.

In order to directly compare our data with recent data published by Tamoutounour *et al* (2013) Immunity 39: 925 and Jakubzick *et al* (2013) Immunity 39: 599, we performed new flow cytometry experiments to analyze expression of monocyte surface molecules on DT-Ly6C+ cells. We find that splenic PBS-Ly6C+ cells express low to negative levels of MHC II, CD11c and F4/80, which is consistent with the published

European Journal of Immunology

data. By comparison, CD64 is significantly up-regulated on splenic and blood Ly6C+ monocytes in DT-treated animals. These data have been included as a revised Figure 2 (results section page 8 paragraph 2).

3) In respect of this subset of monocytes, it should also be noted that most evidence (including Ref#19) indicates that these are derived from Ly6C+MHCIImonocytes in the bloodstream, rather than circulating Ly6C+MHCII+ monocytes as suggested here.

As discussed above, DT-Ly6C+ cells do not express MHC II and we did not intend to suggest that they are derived from circulating MHC II+ cells.

4) For these reasons, it would be important to know how Ly6Chi MHCII- monocyte numbers behave in the bloodstream and spleen after CD11c+ cell depletion. Blood Ly6ChiMHCII- monocytes should also have been included in the microarray experiments.

We agree that the blood Ly6C+ MHC IIneg monocytes were the most appropriate population in the Immgen database to compare to our cells, and this was indeed the population we used for the analyzes shown in Figure 3.

5) It would be important to know whether the expansion of neutrophils seen in the spleen or elsewhere of CD11c cell depleted mice precedes that of monocytes. Not only might that help understand how G-CSF might be relevant to the monocyte expansion, but it would also indicate whether an initial inflammatory reaction might trigger the monocyte accumulation.

This is an important point, and we have extended our work to address this issue. We have now documented the changes in the frequency and numbers of neutrophils in the bone marrow, blood and spleen of PBS- and DT-treated CD11c.DTR mice, and have extended our analyses to 4 days post-depletion of CD11c+ cells. These data demonstrate a concurrent increase in the number of monocytes and neutrophils in the spleen, with a peak accumulation 48 hours after injection of DT. It is, however, impossible to infer a mechanism from these kinetic data to suggest whether there is a causative link between the cell types. Depletion of cells via DT leads to cell death by apoptosis, and we do not expect and inflammatory response to occur in these mice (Miyake *et al* (2007) J Immunol. 178: 5001). These kinetic data have been included in the manuscript (Figure 1 C & D, Supplemental Figure 2 A & B, and results section page 7 paragraph 1).

6) For this purpose, Ly6G should be used throughout the study to identify neutrophils directly.

The emphasis of our study is the monocytosis that occurs in DC-depleted mice. The neutrophilia that occurs upon depletion of DC has previously been well characterized, and for this reason we did not directly identify neutrophils during our study (Tittel *et al* (2009) Nat Method 9: 385; Autenreith *et al* (2011) PLoS Path 8: e1002552; Jiao *et al* (2014) J Immunol 192: 3374). During the initial experiments for this study we used the Ly6G antibody to confirm that we were correctly identifying Ly6Chigh cells as

European Journal of Immunology

monocytes. This confirmed that splenic CD11b+ Ly6Chigh cells were Ly6Gneg monocytes, and the Ly6Cint population was Ly6G+ neutrophils. For clarity, we have now included this information in Figure 1A, and shown the increase in Ly6C+ or Ly6G+ cells in the spleens of DT-treated mice as a graph in Supplemental Figure 2C (results section page 8 paragraph 1).

7) The data on CCR2 are interesting, but require further detail to allow interpretation. For instance, does the CCR2-independent monocyte accumulation occur in other tissues where it is known that this mechanism is needed for monocyte entry in steady state and/or inflammation, such as the intestine, lung, heart, skin? If restricted to the spleen, does this reflect in situ proliferation of pre existing precursors or Ly6C+MHCII+ (or Ly6C+MHCII-) monocytes. BrdU incorporation studies combined with the additional phenotyping indicated above would allow this to be determined.

We did not find an increase in tissue (dermal) monocytes in DT-treated CD11c-DTR mice at the time points tested, and therefore did not focus on the tissues inour CCR2 experiments. We have now included the dermal data in our manuscript (Supplemental Figure 3A). The reviewer makes an important point concerning proliferation of precursors or differentiated monocytes in the spleen of DT-injected mice. We performed a number of BrDU incorporation studies to address this issue and we did not observe differences between cells in PBS- or DT-treated mice. However, there was significant variability in the amount of BrdU incorporation between experiments so we elected not to include these data in the manuscript. The data from these experiments is shown in the Appendix Figure 1.

8) The apparently indirect effect G-CSF is also interesting, but again, its significance and underlying mechanism have not been explored. As noted above, does this suggest that eg neutrophils are needed to produce monocytopoietic factors such as CSF-1, or non-CCR2 dependent chemokines?

As discussed for point 5 above, our data suggest that the expansion of neutrophils does not precede accumulation of monocytes in the spleen, and therefore, it is not clear whether the effect of G-CSF on monocytes is direct or indirect. This point has been enlarged in the discussion (page 19 paragraph 1). While the reviewer raises an interesting question, this study investigates the impact of artificial depletion of CD11c+ cells on the mononuclear phagocyte system, and we feel it is beyond this scope of this work to dissect the underlying mechanisms by which G-CSF promotes expansion of monocytes in this model.

9) In this respect, it would have been interesting to examine the role of CSF-1 in the monocyte accumulation?

As discussed above, this is an interesting question but we feel it is beyond the scope the current manuscript.

10) The gene expression patterns used to conclude that this is a "discrete" population of pro-inflammatory monocytes are rather selective, a reservation underlined by the fact that a modest increase in TNF production is the only functional difference between DTR treated and control spleen monocytes. Most

European Journal of Immunology

monocytes are "poised" for innate inflammatory responses and the majority of the genes which are upregulated are features of differentiation within the monocyte/macrophage lineage. Thus it would be important to know eg how the genes shown in Figure 6B are expressed in dermal macrophages. Even if these do not show complete overlap, it is quite possible that any differences reflect tissue-determined variation rather than "inflammation".

We have performed a gene set enrichment analysis to compare Ly6C+ monocytes to the dermal macrophages populations P4 and P5 published by Tamoutounour *et al* (2013) Immunity 39: 925). We find no enrichment for genes related to TLR pathways or innate immune signaling in dermal macrophage populations, and the false discovery rate (FDR) and family wise error rate (FWER) suggest no relationship with either the TLR or innate immune signaling pathways after p-value adjustments:

Ly6C+ monocytes versus P5 cells Reactome Toll-like receptor cascade P=0.220, FDR = 0.496, FWER = 1.000 Reactome Innate immune system P=0.034, FDR = 0.363, FWER = 1.000

11) The authors do not really discuss what the wider implications of their results are for understanding immune function.

This study documents changes in splenic monocytes following the acute depletion of CD11c+ cells in the CD11c.DTR model and as such we have been cautious about extrapolating our finding to the physiological setting. We believe that it is vital that conclusions made regarding the physiological functions of DC following experimental depletion *in vivo* are also informed by the impact that depletion has on other cells of the mononuclear phagocyte system. However, we have now included a brief discussion of the implications of an elevated population of DT-Ly6C+ monocytes on immune responses in these mice (page 19 paragraph 2).

Reviewer: 2

Comments to the Author

The study by Sivakumaran et al. demonstrates that diphtheria toxin mediated depletion of CD11c+ cells in CD11c-DTR mice leads to a major change in phenotype of Ly-6C+ monocytes in the spleen. These findings are potentially highly relevant to the interpretation of the wealth of studies that have used and continue to use CD11c-DTR mice to ascribe function to dendritic cells (and/or CD11c+ macrophages). While the authors describe aspects of the mechanism leading to generation of ‘variant’ splenic monocytes (eg a role for G-CSF), the underlying cause remains unclear. In this respect, it is important that the authors acknowledge the range of cells directly depleted by DT in these animals (ie. CD11c+ macrophages as well as dendritic cells) that could impact splenic monocyte phenotype. Furthermore, it needs to be determining whether the variant phenotype is restricted to the spleen or is a feature shared by the expanded populations of blood Ly-6C+ monocytes, to understand if this is a splenic micro-environment issue, verify the relationship of expanded blood monocytes to those in the spleen, and ascertain whether

European Journal of Immunology

this study has broader implications when using CD11c-DTR mice to study other tissues (see Major concern 1). I have a number of additional concerns about data interpretation and controls required to ensure validity of the conclusions.

Major concerns:

1. Although the CD11c-DTR mouse was designed to specifically ablate conventional DC, it is clear from numerous publications that administration of DT in this system results in ablation of many tissue macrophages (Rivollier et al. 2012 JEM, Bain et al. 2013 Mucosal Immunol., Landsman et al. 2007 JI). The authors should acknowledge the potential impact of macrophage ablation on the appearance of these 'variant' Ly6C+ monocytes. This is not a trivial point as the authors state that the premise of the study is to interrogate "the impact of DC depletion on other cells of the immune system". In fact, splenic macrophages are known to express CD11c to some degree. Therefore the CD64+ variant monocytes could be cells part-way differentiating to replenishing this population. The authors should include data on the effect of DT administration on F4/80+ CD11b+ splenic macrophages, and of need, the title changed accordingly. (Indeed, Supp. Fig. 1 appears to also show loss of a GFP+ CD11bdull splenic population upon DT treatment – the authors should identify this GFP+ CD11bdull splenic population). The authors should also show if the expanded blood monocytes express the variant phenotype.

As discussed above (Reviewer 1 point 1), the depletion of CD11c+ macrophages from CD11c-DTR mice has been documented, and we have confirmed that splenic macrophages are deleted in our model. These data have been included in Supplemental Figure 1 and we have corrected the text to make it clear that macrophages are also depleted from these mice (page 7 paragraph 1, page 18 paragraph 1). In the light of this point we have changed the title to "Depletion of CD11c+ cells in the CD11c-DTR model drives expansion of unique CD64+ Ly6C+ monocytes that are poised to release TNFα", and have highlighted this point in headline text and in the following areas: the abstract, introduction (page 5 paragraph 3), results (page 7 paragraph 1) discussion (page 18 paragraph 1) and Supplemental Figure 1. As the reviewer points out, there is a population of GFP+ CD11cdull cells in the spleen of CD11c.DTR mice which appear to be depleted upon injection of DT (Supplemental figure 1A). Further analysis shows that these are a heterogeneous group of cells, the majority of which express low levels of CD11b and Ly6C, and therefore are not of myeloid origin (see Appendix Figure 2). We have also extended out phenotyping to blood monocytes and shown they also express the variant phenotype (CD11b+ Ly6Chigh CD64+), although levels of CD64 are lower than in the spleen. These data have been included in the revised Figure 2 (results section page 9 paragraph 1).

2. In Figure 1, the authors should show much more complete gating of different myeloid cells in blood and spleen – this could be Supplementary Figure 1. The authors should show Ly6G expression by the SSCint Ly6C+ CD115int cells to confirm their designation as neutrophils.

All plots shown are gated directly from live (PIneg) cells, as described in the figure legends and methods. We have also included FACS plots showing CD11b+ Ly6Chigh and Ly6C+ cells in the blood in Figure 2D.

European Journal of Immunology

As discussed in response to Reviewer 1 point 6, we have included FACS data in Figure 1 demonstrating that the cells that express lower levels of Ly6C are Ly6G+, confirming their designation as neutrophils.

3. In Figure 2, in order to be able to assess whether these DT-elicited Ly6C+ monocytes express the markers shown in part B, an isotype control needs to be included. From the data shown, it is unclear whether there is any meaningful expression of CD64 by the DT-elicited monocytes. Similarly, CD11c expression by these monocytes appears negligible. Given that CD64 expression is in the title of the manuscript, this must be addressed.

As discussed for Reviewer 1 point 2, we have included data using isotype controls to demonstrate the relative levels of expression of different surface molecules on DT-Ly6C+ cells. These data show that MHCII, CD11c and F4/80 are all expressed at very low to negative levels on splenic monocytes. By comparison CD64 is significantly up-regulated on Ly6C+ monocytes in the blood and spleen of DT-treated mice. For this reason, we feel it is an appropriate marker to define our cells phenotypically.

4. According the text, the axes in Figure 4 are labelled incorrectly. Also, it is unclear what the graph in 4B is showing. According to the legend, it shows "the frequency of WT or Ccr2–/– cells within the CD11b+ Ly6C+ monocyte population". If this is correct, what is making up the other 99% of cells? Part C is said to represent numbers but the axis is %.

The reviewer correctly identified errors in the labeling for Figure 4. The Y axis on the graph for Figure 4C has been corrected and this figure has been reviewed to make the data clearer (legend page 41 paragraph 1).

5. In Figure 5, treatment with exogenous G-CSF is purported to 'expand' and 'mobilize' a population of Ly6C+ monocytes that are transcriptionally indistinguishable from DT-Ly6C+ monocytes. The transcriptomic data presented supports the conclusion that G-CSF causes 'differentiation' of monocytes to the DT-Ly6C+ phenotype, but no data is provided that shows the number of monocytes is 'expanded' or monocytes are 'mobilized' – please show the data for numbers in the blood and spleen or change the text accordingly.

As requested we have now included a figure showing the expansion in the numbers of monocytes and neutrophils in the spleens of mice treated with G-CSF (Figure 5C; results section page 13 paragraph 2).

6. In Figure 7 the authors test the production of cytokines in response to LPS stimulation. They show TNFα and IL6 and state "no significant differences were observed for the other cytokines". The authors should indicate some of the other cytokines measured. Analysis of the kinetics of the appearance, persistence and contraction of DT-elicited Ly6C+ monocytes (Point 4) will allow the reader to assess the functional consequences of the presence of monocytes with heightened responsiveness to TLR stimulation.

European Journal of Immunology

We have now included graphs showing the data from the other cytokines tested as Figure 6C (page 15 paragraph 1). The reviewer makes an important point about understanding the kinetics of monocyte expansion in this model, and we have now included additional data to address this issue (see also Reviewer 1 point 5; results section page 7 paragraph 1 and Figure 1C and D). Monocytes accumulate in the spleen and blood 48 hours after injection of DT, and are decreasing 4 days post-injection, at which point repopulation of DC has occurred. Therefore, these data suggest a defined window in which DT-Ly6C+ may contribute to an innate immune response in the absence of DC. This point has been discussed in the text (page 19 paragraph 2).

7. In the Discussion, the authors claim to have identified a population of monocytes in spleen that "spontaneously express proteins associated with entry into tissues" – this reviewer is not entirely clear what markers the authors are referring to. Ly6Chi monocytes arriving in the dermis of the skin or in the intestinal mucosa begin to downregulate Ly6C and CCR2, and upregulate MHCII – DT-elicited Ly6C+ monocytes have high levels of Ly6C and CCR2, and fail to express MHCII. Therefore the phenotypic similarity between DT-elicited cells and recently extravasated monocytes in tissues is limited. Because the authors place such emphasis on CD64 expression, it would be informative if they compared the expression levels of CD64 on DT-Ly6C+ monocytes with monocytes in the dermis, or at least a representative tissue macrophage to allow one to establish the extent of CD64 expression.

The reviewer makes an important point here and we rewritten our discussion to remove the emphasis on a link between DT-Ly6C+ cells and dermal monocytes (page 17 paragraph 2). Expression of CD64 is associated with the extravasation of monocytes into tissues, and for this reason we compared our DT-Ly6C+ cells with dermal monocytes for this manuscript. However, unlike tissue monocytes, our cells express high levels of Ly6C and low levels of MHC II. Our data show that, despite elevated expression of CD64, DT-Ly6C+ cells most closely resemble splenic monocytes at the transcriptional and phenotypic level. We have also highlighted this point in the results section (page 11 paragraph 1). Comparison of the level of CD64 expression on splenic and dermal monocytes shows that CD64 expression on splenic DT-Ly6C+ cells is lower than on cells in the dermis of DT-treated mice. We have included these data in a new Supplemental Figure 3.

Minor concerns:

1. In the Introduction the authors state that 'Classical' or 'inflammatory' Ly6C+ monocytes are released from the BM in response to infection via the ligation of the chemokine receptor CCR2". This sentence is misleading as it suggests that these monocytes are only elicited by infection, whereas it is clear that these monocytes are released continuously into the blood of unmanipulated mice.

To clarify the point that monocytes are released from the bone marrow (BM) in the absence of infection, we have simplified the sentence to read "Classical or inflammatory Ly6C+ monocytes released from the BM via ligation of the chemokine receptor CCR2". (Page 4 paragraph 2).

2. On page 9, the authors state "The heat map in Figure 3B shows an incremental increase in the expression level of most of the genes tested in the progression to DT-Ly6C+ monocytes" – this is unclear.

We have re-written the sentence to describe the data in Figure 3B more clearly. The sentence now reads "The heat map in Figure 3B shows the relative expression of the panel of genes by MDP, cMoP and Ly6C+ monocytes from PBS- and DT-treated mice. These data demonstrate that DT-Ly6C+ monocytes express transcription factors associated with monocyte differentiation at higher levels than the other cell populations…" (Page 10 paragraph 1).

Reviewer: 3

Comments to the Author

In the paper entitled " Depletion of dendritic cells in the CD11c-DTR model drives expansion of unique CD64+ Ly6C+ monocytes that are poised to release TNFα, Sivakumaran and colleagues have attempted to better characterize the monocyte influx observed upon DT injection on CD11c-DTR mice.

1. As shown on figure 1 and as previously published, the authors observed an increase in frequency of monocytes as well as neutrophils 48h after injection of DT in CD11c-DTR mice. As the authors mainly focused on the spleen, and considering that in the original paper describing the CD11cDTR mice (Jung et al, Immunity, 2002) it was mentioned that some T cells may express CD11c and thus might be depleted upon DT treatment, the authors should maybe provide an overall study of the consequences of DT treatment on all cell types within the spleen (B cells, CD4+ T cells, CD8+ T cells, DC, monocytes, neutrophils, macrophages).

As discussed in reply to Reviewer 1 point 1, other immune cells express CD11c and have the potential to be depleted from CD11c-DTR mice. This has been previously investigated in this and other CD11c-specific depletion models Probst *et al* (2005) Clin Exp Immunol 141: 398; Hochweller et al (2008) Eur J Immunol 38: 2776). We investigated depletion of T cells, B cells, NK cells and macrophages in our model. We did not observe a change in the frequency of lymphoid populations, but CD11b+ F4/80+ macrophages are depleted from the spleen as described previously (Hochweller et al (2008) Eur J Immunol 38: 2776). We have included these data in Supplemental Figure 1.

2. This influx of monocytes and neutrophils could be seen as a sign of inflammation. As it is not observed with Langerin-DTR mice (by the way those results should be shown) in which the cell depletion within the spleen might be too subtle to trigger any inflammatory call, it would be of great interest to study if other DTR mouse model with significant cell depletion within the spleen might induce similar monocyte and neutrophil recruitment or if this phenotype is uniquely particular to the CD11c-DTR mouse model.

We have included the data from the Langerin-DTR mice in Supplemental Figure 1, demonstrating that depletion of Langerin+ cells does not lead to monocytosis. As discussed in reply to Reviewer 1, point 1,

depletion of DC from other DTR models has also been reported to result in monocytosis and is not specific to this mouse model (reviewed by van Blijswijk *et al* (2013) Eur J Immunol. 43: 22). In particular, use of the transcription factor Zbtb46 to express the DTR in DC but not monocytes or macrophages also resulted in an increase in the percentage of splenic monocytes after injection of DT, to a greater extent than that seen in the CD11c-DTR mice (Meredith *et al* (2012) J Exp Med 209: 1153). In addition, cell death in the DTR model occurs by apoptosis and does not induce an inflammatory response (Miyake *et al* (2007) J Immunol. 178: 5001). Therefore, we believe that our data strongly support that hypothesis that loss of DC *per se* in the CD11c-DTR model, and not inflammation, drives expansion of monocytes and neutrophils. We have discussed this point in the text (page 8 paragraph 1 and page 18 paragraph 1).

3. As presented on figure 1, less than 30 h post DT treatment, the percentage of monocytes in the spleen are significantly increased whereas the absolutenumbers are unchanged. Nevertheless, 24h later this increase is significant for the frequency as well as the absolute numbers. 48h post DT treatment, the monocyte frequency within the BM is significantly increased whereas the absolute numbers are unchanged. Did the authors checked if later on the absolute numbers within the BM might be significantly increased as it happened with the spleen? Could the authors discuss this issue ?

We have now included an extended kinetic analysis in the paper (Figure 1C & D and Supplemental Figure 2A and B). At the early time point we believe that the change in frequency, but not number, of monocytes in the spleen reflects the loss of DC and macrophages, with only a small increase in monocytes. However, at 48 hours the on-going increase in monocytes leads to a significant accumulation of cells in the spleen. In the bone marrow and blood we find that monocytes do not start to increase until 2 days post-injection of DT, but continue to increase over time. These data have been discussed in the manuscript (results section page 7 paragraph 1).

4. A presented on figure 2, those monocytes appeared to have fragmented nucleus and slightly more vacuoles and they slightly increased their expression of CD64 and F4/80 which are typical macrophage markers. Interestingly, those monocytes do not upregulate CD11c and MHCII. Altogether, the phenotype changes appeared quite subtle. It would be interesting to also complete with the analysis of neutrophils (morphology and surface-marker phenotype) as they are also increased in numbers and later on in the paper the authors will assess if those cells could be related to neutrophils. Moreover, it would be interesting to compare side by side CD64 and F4/80 expression by DT-Ly6C+ as well as tissue macrophages and inflammatory monocytes observed in Listeria or toxoplasma infected mice.

The increase in neutrophils that occurs upon disruption of the DC niche has been extensively characterized in a number of publications, while the change in monocytes has been largely ignored (Tittel *et al* (2009) Nat Method 9: 385; Autenreith *et al* (2011) PLoS Path 8: e1002552; Jiao *et al* (2014) J Immunol 192: 3374). For this reason we have focused our research on the monocytosis that

European Journal of Immunology

occurs in DC-depleted mice and have not repeated an analysis of the neutrophils. We have now directly compared the expression of CD64 on splenic and dermal monocytes in DT-treated mice, as discussed for Reviewer 2 point 7 (Supplemental Figure 3B). We agree that it would be interesting to compare the phenotype of DT-Ly6C+ monocytes, which arise in the absence of inflammation, to inflammatory monocytes in infected mice. However, we feel that these experiments are beyond the scope of the current manuscript.

5. According to Figure 3D, DT Ly6C+ monocytes clustered with PBS Ly6C+ monocytes and are also closely related to Dermis-P1 and dermis-P2 which are highly related to CCR2-dependent blood monocytes Thus the use of competitive BM chimera combining WT and Ccr2-/- bone marrow was absolutely required in this study. Unfortunately, the authors do not seem to exploit properly the results. First of all, the Ccr2-/- are supposedly CD45.2+ and thus the WT should be followed as CD45.2+CD45.1+ and the Ccr2-/- should be CD45.2+ whereas on the figure 4, the Ccr2-/- are followed as CD45.1+ CD45.2.
The schematic for Figure 4A was indeed mis-labelled and has now been corrected (legend page 41).

6. The monocytes analysed in PBS should be considered as conventional monocyte and thus CCR2 dependent. Thus in the chimera mice, the ratio of 71.8% versus 27.2% observed on Figure 4B should be interpreted as the ratio of CCR2 dependent monocyte and should also be the ratio observed for blood monocyte whereas other cells within the blood such as B cells or neutrophils should have a 50-50 ratio. As a consequence, the ratio observed in DT treated mice (71.2% versus 27.7%) should be seen as CCR2-dependency of those DT Ly6C+ cells. Moreover figure 4C should be taken carefully as Ccr2-/- mice have definitely some leakiness and still have some monocytes within the periphery. The ratio no DT /DT seems to be similar between WT and Ccr2-/- mice. Thus those DT Ly6C+ cells might definitely be considered as Ccr2 dependent and thus of monocyte origin.
The reviewer makes an important point about the careful interpretation of the results from the competitive chimera experiments. However, we do not agree that the ratio of WT:KO monocytes observed in the spleen should be considered the same as that in the blood because of the contribution of extramedullary monocytes to the pool of cells in the spleen (Swirski *et al* (2009) Science 325: 612). This means that there should be more contribution from *Ccr2*-/- cells to splenic monocytes than is seen in the blood. As the reviewer describes, the ratio of WT:KO neutrophils in the spleen was approximately 1:1 (Appendix Figure 3). We strongly believe that our data demonstrate independence from CCR2 by DTLy6C + monocytes. First, we have shown that splenic Ly6C+ monocytes are increased in the spleens of *Ccr2*-/- mice. We agree that there is some leakiness in this model, however the fold increase in monocytes is equivalent, if not enhanced, in the spleen of CD11c.DTR.*Ccr2*-/- animals compared to controls. Second, we directly compared the expansion of cells that could or could not be recruited from the BM via CCR2 using competitive chimeras in which developing monocytes were derived from wild-type or *Ccr2*-/- donors. Again, these data clearly demonstrate that cells derived from *Ccr2*-/- donors can contribute to

European Journal of Immunology

splenic monocytosis on depletion of CD11c+ cells. We have re-plotted the data from the competitive chimera experiments to make it clearer for the readers (Figure 4C).

Together these data strongly suggest that splenic DT-Ly6C+ monocytes contain cells that have not required signaling via CCR2 for expansion in DC-depleted animals. We agree that the DT-Ly6C+ cells are of monocyte origin (as supported by our bioinformatics data), but would argue that these cells may be derived directly from the spleen and are not recruited from the bone marrow.

7. Regarding the data presented on Figure 5, it would be important to include the control consisting of PBS + αG-CSF to compare with DT + αG-CSF. The comparison with the neutrophils presented on Figure 5D should be done using neutrophils isolated from DT treated spleens.

The experiments presented in Figure 5 were designed to investigate whether GCSF was required for the DT-induced increase in splenic monocytes observed in CD11c.DTR mice. Therefore, we decided that the important control in these experiments was to compare the effect of administering αG-CSF neutralizing antibodies with an isotype control in the DT-treated group. For this reason, we did not include a group of PBS-treated control animals that received αG-CSF antibodies.

The aim of the experiment presented in Figure 5D was to investigate whether DT-Ly6C+ cells shared a transcriptional profile with neutrophils because recent data has described trans-differentiation of monocytes from neutrophils (Köffel et al (2014) Blood 124: 2713). We did not isolate neutrophils from DT-treated mice for this experiment because, as discussed above, neutrophils are not the focus of this work and are beyond the scope of this manuscript.

8. The comparison presented on Figure 5C should be completed with splenic monocytes isolated from listeria or toxoplasma infected mice as those cells might be related to the inflammatory monocytes known to produce TNF as presented on figure 6.

As discussed above, these experiments are interesting but are beyond the scope of this manuscript.

9. At the end of this paper, I am not sure to be completely convinced that those DT Ly6Chigh cells are not related to inflammatory monocytes. The authors mentioned in the discussion that they display many characteristics associated with Ly6Cneg monocytes but they did not really compared their signature.

It is not really clear to me neither if those cells are expanding extramedullary (Fig 1D) and independently of CCR2.

This manuscript describes the transient accumulation of a population of CD11b+ Ly6Chigh monocyte-like cells that express CD64 but not other molecules associated with inflammation or activation (e.g. CD11c, MHC II). Transcriptional profiling demonstrated that these cells are most similar to classical Ly6Chigh monocytes, and do not co-locate with Ly6Cneg in our multi-dimensional scaling analysis (Figure 3A), despite the expression of some molecules associated with Ly6Cneg monocytes (Figure 3B). However, as discussed for Reviewer 1 point 11, we have been cautious about specifically categorizing these cells within myeloid lineages since they arise after the artificial acute depletion of DC in this model.

European Journal of Immunology

As discussed in response to point 6 above, we strongly believe that some Ly6C+ monocytes are expanding in the spleen independently of CCR2.

10. Finally, the authors did not really prove that those iNOS producing monocytes were immunospppressive and they did not provide any proof that any conclusions made with the CD11cDTR mice upon DT treatment was not likely due to the depletion of DC but rather to the presence of those Ly6C+ cells in the spleen or even to the neutrophils.

We have recently published a collaborative paper in Science Translational Medicine (D'Aveni *et al* (2015) 7: 281) in which we show that G-CSF drives the expansion of monocytes that produce iNOS in the context of a model of graftversus-host disease. This is a different study and highlights that the functional outcome of monocytosis may be context dependent.

Minor points:

Significant number of mistakes within the figure legends:

Fig1A: it is starting by "left" whereas right and left are concerned

Fig1B right and left are inverted; It is repeatedly written DTR-treated recipients instead of DT-treated recipient

Fig1D Why not providing the exact number of mouse used to calculate the statistics instead of n=14-19 and n=11-16

Figure 1 has been extensively revised to include the kinetic experiments and the legend has now been checked and re-written to reflect the new figure.

Fig2A Geisma instead of Giemsa

This typo has been corrected (page 39).

Fig2C: why did the authors use different gating strategy to follow the potentially similar (or not) cells: CD11b+Ly6Chigh or CD11b+Ly6ChighCD115high

The change in staining panels reflected the evolution of the project, and depended on which other markers were being measured in the same experiment. During this work a number of seminal publications led to us improving our staining panels for flow cytometry (e.g. Tamoutounour *et al* (2013) Immunity 39: 925). The results using the different panels were consistent and use of these different panels does not change the interpretation of the experiments.

Fig4B: not only the axe labels might be wrong (CD45.1 / CD45.2) but also the plot representing the chimerism gives a percentage with values below 0.8. Can the frequency be below 0.8% or the authors meant 80 % ?

We appreciate that the Reviewer spotting these errors. They have now been corrected and the legend has been re-written (page 41).

European Journal of Immunology

Fig4C: the legend says that the graph shows the numbers of CD11b+Ly6C+ whereas on the figure itself it is labeled as percentage.

We have now corrected the mis-labelled axis to show the number of cells.

Instead of mentioning "statistical analyses were carried out with a Student's unpaired T-test or Mann Whitney as appropriate, could the authors specify which statistical test they used for each plot. This is an important information for the reader.

We have now used different symbols in the figures to demonstrate whether the data was analyzed using a Student's T-test or Mann Whitney test. This is listed in the legend for every figure.

Appendix

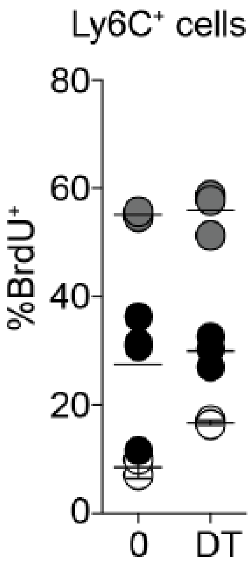

Appendix Figure 1. Graph showing the frequency of BrdU positive CD11b^{+} Ly6C^{high} monocytes in the spleen of PBS- or DT-treated mice. CD11c-DTR mice were injected with PBS or DT and were sacrificed 48 hours later. Mice received an i.p. injection of BrdU 12 hours before take-down. Colors represent 3 independent experiments, circles represent individual mice.

European Journal of Immunology

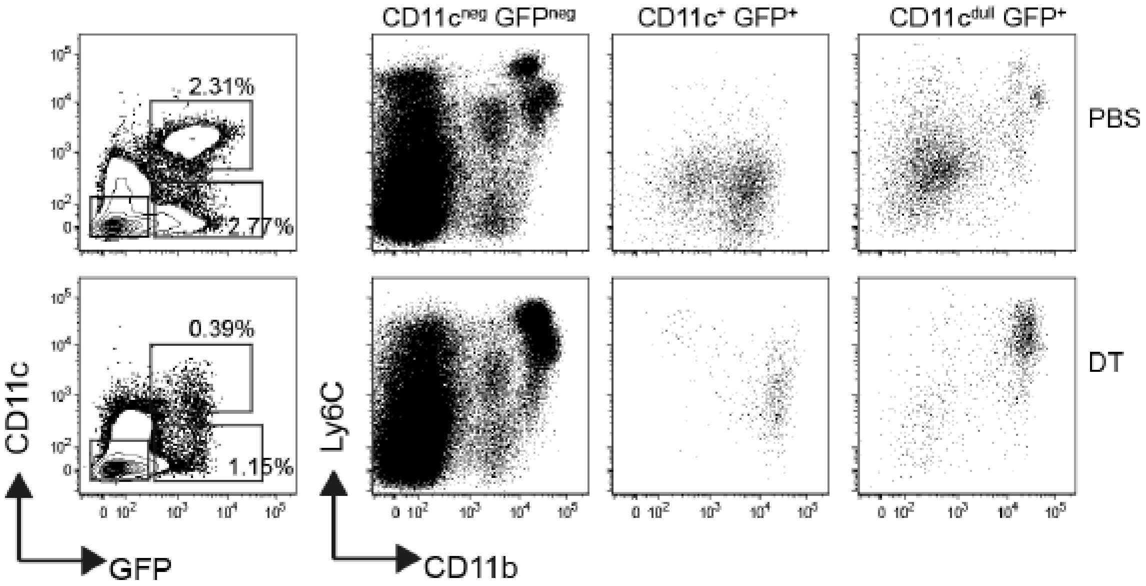

Appendix Figure 2. CD11c-DTR mice were injected with PBS or DT and sacrificed 48 hours later. *Left* - representative contour plots showing the expression of CD11c and GFP on live cells. *Right* - representative dot plots showing the expression of CD11b and Ly6C by cells in the different gated populations.

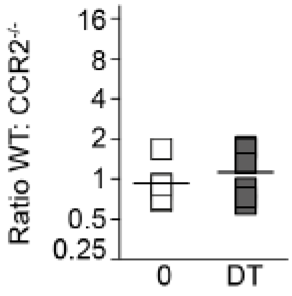

Appendix Figure 3. CD11c.DTR.CD45.1 recipients were lethally irradiated and reconstituted with an equal mixture of BM cells isolated from *Ccr2*$^{-/-}$ .CD11c.DTR.CD45.2 or *Ccr2*$^{+/+}$.CD11cDTR.CD45.1.2 donors. Eight weeks later, chimeras were injected with DT, and expansion of *Ccr2*$^{-/-}$ (CD45.2) versus WT (CD45.1.2) CD11b^{+} Ly6C^{int} neutrophils compared in the spleen. The graph shows the relative contribution of WT or CCR2$^{-/-}$ cells to the neutrophil population.

Peer review correspondence

European Journal of Immunology

Second Editorial Decision – 8 September 2015

Dear Dr. Bennett,

Thank you for submitting your revised manuscript ID eji.201545789.R1 entitled "Depletion of dendritic cells in the CD11c-DTR model drives expansion of unique CD64+ Ly6C+ monocytes that are poised to release TNFα" to the European Journal of Immunology.

Your manuscript has been re-reviewed and the comments of the referees are included at the bottom of this letter. Although the referees have recommended publication, some revisions to your manuscript have been requested. Therefore, I invite you to respond to the comments of the referee #1 and revise your manuscript accordingly.

You should also pay close attention to the editorial comments included below.

If the revision of the paper is expected to take more than three months, please inform the editorial office. Revisions taking longer than six months may be assessed by new referee(s) to ensure the relevance and timeliness of the data.

Once again, thank you for submitting your manuscript to European Journal of Immunology. We look forward to receiving your revision.

Yours sincerely,
Laura Soto Vazquez

on behalf of Prof. Bernard Malissen

Editorial Office
European Journal of Immunology
e-mail: ejied@wiley.com
www.eji-journal.eu

****

Reviewer: 1

Comments to the Author

European Journal of Immunology

The authors have added new information and several clarifications to address my original comments, thus improving this manuscript to a substantial extent. Some points arising from the new material need attention:

1) The 'discrete' population of monocytes that expands in the spleen after depletion of CD11c+ cells has now been characterised in more detail, but some issues remain concerning their origin. For instance, it is unclear how their numbers expand so much, when the process is said not to be reflect recruitment of monocytes from the bloodstream, but the BrdU data provided would argue against local proliferation, which is the most obvious alternative mechanism. The absence of recruitment is based primarily on the experiments on CCR2, which are actually not as clear as the authors maintain. Indeed the revised annotation of Figure 4C seems to suggest that the absolute expansion of CCR2-/- monocytes is much reduced compared with that of WT monocytes. Furthermore, the transcriptional analysis in Figure 2B indicates that expression of CCR2 mRNA is higher amongst the expanded monocyte population. Given that a simple interpretation of the transcriptional and otherdata remains the possibility that the CD64+ monocytes are a differentiation stage within conventional monocyte development, the conclusions about the expanded cells being a locally derived discrete population are still not justified.

2) Figure 1B is not cited in the text, while the right panel of Figure 1D is not annotated properly.

3) Figure 1A shows a large population of Ly6Glo/- Ly6cint cells in DT treated spleen whose identity is not discussed.

4) The shifts in MHCII and CD64 expression shown in Figure 2 are rather small, even allowing for the significant increase in MFIs.

5) Although not labelled as statistically significant, the additional cytokine data provided in Figure 6B suggest that TNFa production is not the only feature of the expanded monocytes, as IL6 and GM-CSF appear to be upregulated almost as much.

6) The authors' example of a biological context for monocytes of this kind is the possibility that they may be immunosuppressive in GvHD via increased expression of iNOS. However this does not appear to be an over-expressed gene in the population examined here and NO production has also not been measured.

Reviewer: 2

Comments to the Author

The authors have addressed most of the points raised in the review process. The paper is interesting and relevant to the interpretation of the wealth of studies that have used and continue to use CD11c-DTR mice to ascribe function to dendritic cells and CD11c+ macrophages.

Reviewer: 3

Comments to the Author

In the revision version of the paper entitled "Depletion of CD11c cells in the CD11c-DTR model drives expansion of unique CD64+ Ly6C+ monocytes that are poised to release TNFa, the authors have correctly answered all the points raised and have significantly improved the quality of the manuscript.

Second revision – authors' response – 10 September 2015

Reviewer: 1

Comments to the Author

1) The 'discrete' population of monocytes that expands in the spleen after depletion of CD11c+ cells has now been characterised in more detail, but some issues remain concerning their origin. For instance, it is unclear how their numbers expand so much, when the process is said not to be reflect recruitment of monocytes from the bloodstream, but the BrdU data provided would argue against local proliferation, which is the most obvious alternative mechanism. The absence of recruitment is based primarily on the experiments on CCR2, which are actually not as clear as the authors maintain. Indeed the revised annotation of Figure 4C seems to suggest that the absolute expansion of CCR2-/- monocytes is much reduced compared with that of WT monocytes. Furthermore, the transcriptional analysis in Figure 2B indicates that expression of CCR2 mRNA is higher amongst the expanded monocyte population. Given that a simple interpretation of the transcriptional and other data remains the possibility that the CD64+ monocytes are a differentiation stage within conventional monocyte development, the conclusions about the expanded cells being a locally derived discrete population are still not justified.

We contend that the Ly6C+ monocytes that arise in the spleen of mice depleted of CD11c+ cells can be considered a locally-derived monocyte population for the following reasons:

1. The experiments shown in Figure 4 using both Ccr2-/- mice and competitive chimeras both demonstrate an expansion of Ly6C+Ccr2-/- cells in the spleens of DT-treated mice. The magnitude of expansion is comparable between the experiments: in Figure 4A Ly6C+ cells expand 2.1 fold after DT-treatment of CD11c.DTR.Ccr2+/+ mice compared to 2.7 fold in CD11c.DTR.Ccr2-/-; in Figure 4C WT Ly6C+ cells expand 2.9 fold after DT treatment compared to an expansion of 1.9 fold for Ccr2-/- cells in the same mouse. These data have been included in the Results (page 13, paragraph 1). As the reviewer points out, Ccr2 mRNA is more highly expressed by DT-Ly6C+ cells, but expression of the gene alone does not indicate whether this receptor is required or used by these cells following injection of DT.

2. While we observed a similar pattern of expansion of monocytes in the BM and blood, suggesting continuity of cells between these compartments, the kinetics of monocytosis in the spleen were distinct. Ly6C+ cells began to accumulate 1 day after injection of DT, and peaked on day 2, preceding accumulation of these cells in the BM and blood.

European Journal of Immunology

3. We agree that the mechanism of expansion of Ly6C+ monocytes in the spleen is unclear. We did not detect an increase in proliferation of differentiated Ly6C+ monocytes in the spleen of DT-treated mice (previously attached as an appendix to our reply to the reviewers); however, this does not exclude the possibility that Ly6C+ monocytes and/or their precursors are expanding directly in the spleen. In these experiments, we attempted to identify the cMoP population that has been reported to be present in the spleen, but were not able to do so (Hettinger *et al* (2013).Nat Immunol 14: 821). Therefore, we cannot exclude the possibility that a monocyte precursor is proliferating in the spleen after injection of DT. In addition, our kinetic experiments demonstrate accumulation of splenic Ly6C+ cells 1 day after injection of DT, and it is possible that we have missed the point of proliferation in our BrdU experiments. We have now included these caveats within the Discussion (page 18, paragraph 2).

2) Figure 1B is not cited in the text, while the right panel of Figure 1D is not annotated properly.
Figure 1B has now been cited in the text (page 7, paragraph 1), and the legend by the right panel for Figure 1D has been corrected.

3) Figure 1A shows a large population of Ly6Glo/- Ly6cint cells in DT treated spleen whose identity is not discussed.
As the reviewer points out, the FACS plots showing the expression of Ly6C and Ly6G by Ly6C+ monocytes reveal the presence of a population of Ly6Gneg Ly6Cint cells in DT-treated mice. These cells fell within the Ly6Cint “neutrophil” gate that we used in this study, and therefore were not included in subsequent analyzes. We have included reference to this population in the Results (page 7, paragraph 1).

4) The shifts in MHCII and CD64 expression shown in Figure 2 are rather small, even allowing for the significant increase in MFIs.
We did not propose that the change in MHCII expression is meaningful, and inclusion of an isotype control demonstrated that splenic Ly6C+ monocytes only expressed background levels of MHCII. By contrast, CD64 is expressed above background by Ly6C+ cells in both PBS- and DT-treated mice, and the mean MFI for CD64 increases 2 fold from 897.4 ±59.38 (SEM) on PBS-Ly6C+ cells to 1805 ±108.8 on DT-Ly6C+ cells. We have clarified this issue in the Results (page 9, paragraph 1).

5) Although not labelled as statistically significant, the additional cytokine data provided in Figure 6B suggest that TNFa production is not the only feature of the expanded monocytes, as IL6 and GM-CSF appear to be upregulated almost as much.
We agree with the Reviewer that a similar trend exists, but are reluctant to make strong statements about the production of IL-6 and GM-CSF because the differences are not statistically significant.

6) The authors' example of a biological context for monocytes of this kind is the possibility that they may be immunosuppressive in GvHD via increased expression of iNOS. However this does not appear to be an over-expressed gene in the population examined here and NO production has also not been measured.

We have recently published a collaborative paper in Science Translational Medicine (D'Aveni *et al* (2015) 7: 281) in which we show that G-CSF drives the expansion of monocytes that produce iNOS in the context of a model of graftversus-host disease. This is a different model system and we included the reference to highlight our suggestion that the functional outcome of monocytosis may be context-dependent. There have equally been a number of papers demonstrating that monocytes are pathological when they expand within an inflammatory environment (e.g. Grainger *et al* (2013) Nat Med 19: 713-721).

Third Editorial Decision – 24 September 2015

Dear Dr. Bennett,

It is a pleasure to provisionally accept your manuscript entitled "Depletion of dendritic cells in the CD11c-DTR model drives expansion of unique CD64+ Ly6C+ monocytes that are poised to release TNFα" for publication in the European Journal of Immunology. For final acceptance, please follow the instructions below and return the requested items as soon as possible as we cannot process your manuscript further until all items listed below are dealt with.

Please note that EJI articles are now published online a few days after final acceptance (see Accepted Articles: http://onlinelibrary.wiley.com/journal/10.1002/(ISSN)1521-4141/accepted). The files used for the Accepted Articles are the final files and information supplied by you in Manuscript Central. You should therefore check that all the information (including author names) is correct as changes will NOT be permitted until the proofs stage.

We look forward to hearing from you and thank you for submitting your manuscript to the European Journal of Immunology.

Yours sincerely,
Karen Chu

on behalf of Prof. Bernard Malissen

Dr. Karen Chu
Editorial Office

Peer review correspondence

European Journal of Immunology
e-mail: ejied@wiley.com
www.eji-journal.eu
